# Ketones and Sleep: A Narrative Review of Mechanisms, Preclinical Models, and Human Evidence

**DOI:** 10.3390/nu18203437

**Published:** 2026-10-20

**Authors:** Jack C. Jensen, Parker C. Leavitt, Christa J. Nehs

**Affiliations:** 1 Mass General Brigham Department of Anesthesiology, Massachusetts General Hospital, Charlestown, MA 02129, USA; jcjensen29@gmail.com (J.C.J.); parkerleavitt29@gmail.com (P.C.L.); 2 Division of Sleep Medicine, Harvard Medical School, Boston, MA 02155, USA; 3 Metabolic and Mental Health Program, McLean Hospital, Belmont, MA 02478, USA

**Keywords:** beta-hydroxybutyrate, acetoacetate, ketogenic diet, exogenous ketones, sleep architecture, slow-wave sleep, REM sleep, circadian rhythm, inflammation, obstructive sleep apnea

## Abstract

Background/Objectives: Sleep is a metabolically dynamic state, and ketone bodies may interact with sleep physiology as both oxidative substrates and signaling metabolites. β-Hydroxybutyrate (BHB), acetoacetate (AcAc), and acetone rise during fasting, carbohydrate restriction, ketogenic diets used for therapeutic or non-therapeutic purposes, prolonged exercise, selected pathological states of insulin deficiency, and after consumption of exogenous ketones. This narrative review synthesizes mechanistic, preclinical, and human evidence linking endogenously generated ketosis and ketosis generated from exogenous ketone consumption to sleep architecture, sleep quality, sleep-disordered breathing, and sleep-relevant metabolic pathways. Methods: We reviewed studies involving ketogenic diets, fasting or caloric restriction, and consumption of exogenous ketones, prioritizing investigations with polysomnography, electroencephalography (EEG), sleep architecture, respiratory, or validated sleep-questionnaire outcomes, while also considering mechanistic studies of neuronal excitability, inflammation, epigenetic regulation, circadian timing, and brain energetics. Results: The most direct preclinical evidence indicates that AcAc, but not BHB, increases slow-wave activity (delta power 0.5–4 Hz) during non-rapid eye movement (NREM) sleep and suppresses glutamate release in mice, supporting a potential role for ketone metabolism in sleep homeostasis. Dietary, fasting, and aging models further suggest that lipid/ketone metabolism can alter NREM/rapid eye movement (REM) balance, circadian metabolic rhythms, and sleep–wake responses to energetic stress, with aged-rat data showing sex-specific REM and cognitive effects of ketogenic interventions. Human studies are suggestive but heterogeneous: acute carbohydrate restriction can increase slow-wave sleep and reduce REM sleep in healthy adults; ketogenic therapies may improve subjective sleep in selected neurological and metabolic populations; and ketone ester ingestion can improve sleep efficiency after strenuous evening exercise, but not during hypoxia-induced sleep disruption. Conclusions: Ketones are sleep-relevant metabolic signals, but their effects are conditional rather than universally sleep-promoting. They may also indirectly affect sleep by enhancing wakefulness quality. The ketone concentration range associated with optimal sleep outcomes remains undefined, and potential dose-, timing-, and context-dependent effects on sleep require further study. Future studies should distinguish AcAc from BHB biology, measure nocturnal ketone dynamics, incorporate PSG/EEG spectral endpoints, and separate ketosis-specific effects from weight loss, energy deficit, exercise load, respiratory physiology, circadian timing, and disease-related confounders.

## 1. Introduction

Sleep reduces some energy-consuming functions while enabling other metabolically demanding maintenance functions in a stage-specific manner. Across wakefulness, non-rapid eye movement (NREM) sleep, and rapid eye movement (REM) sleep, the brain changes substrate use, neuromodulatory tone, autonomic output, immune signaling, and transcriptional timing [1]. During NREM sleep, many global indices of energy demand decline, yet this reduction in outward activity coincides with active cellular and systems-level processes, including substrate redistribution, synaptic remodeling, cellular repair, immune regulation, neuroendocrine signaling, and interstitial solute clearance. REM sleep further complicates this picture because selected neural circuits display wake-like activation despite peripheral muscle atonia. This metabolic plasticity creates a plausible interface between sleep regulation and nutritional state, particularly for metabolites that can simultaneously support cellular energetics and modify signaling pathways. Ketone bodies—principally beta-hydroxybutyrate (BHB), acetoacetate (AcAc), and acetone—are generated during fasting, carbohydrate restriction, prolonged exercise, and ketogenic dietary therapy, and can also rise acutely after ketone ester or ketone salt ingestion. Their relevance to sleep biology is therefore not limited to alternative fuel provision; ketones also regulate cell signaling, redox state, inflammation, epigenetic marks, and neuronal excitability [2,3,4]. Thus, ketones may influence sleep not merely by supplying energy but by modulating the metabolic and signaling programs through which sleep supports neural and systemic homeostasis.

The question for clinicians is not simply whether ketosis improves sleep. Existing data support a more nuanced formulation: ketone availability may alter sleep depth, REM/NREM balance, sleep efficiency, respiratory physiology, or daytime sleepiness depending on ketone species, route of induction, circadian timing, sleep pressure, inflammatory tone, body composition, exercise load, sex, age, and disease context. This review therefore separates mechanistic and preclinical data from human dietary, exogenous-ketone, neurological, metabolic, and respiratory studies and emphasizes where evidence is direct versus inferential.

## 2. Materials and Methods

The objective of this narrative review was to assemble the currently identifiable literature on ketones and sleep, with special emphasis on direct sleep outcomes. PubMed was searched from database inception through September 2026 for literature relevant to ketone metabolism and sleep, yielding approximately 329 records. Searches combined ketone-related terms (e.g., “ketogenic diet,” “ketosis,” “beta-hydroxybutyrate” or “BHB,” “acetoacetate” or “AcAc,” “ketone ester,” “ketone salt,” “fasting,” and “very-low-carbohydrate diet”) with sleep-related terms (e.g., “sleep,” “polysomnography,” “EEG,” “sleep architecture,” “NREM,” “REM,” “slow-wave sleep,” “slow-wave activity,” “insomnia,” “sleep quality,” “daytime sleepiness,” “sleep apnea,” “obesity hypoventilation,” and “circadian rhythms”). Targeted searches were also performed for mechanistic pathways relevant to ketone–sleep interactions, including “glutamate,” “KATP,” “adenosine,” “NLRP3,” “HDAC,” “beta-hydroxybutyrylation,” and “brain energetics.” Titles and abstracts were screened for relevance, followed by full-text review of potentially relevant articles. Reference lists of relevant primary studies and reviews were also screened, and additional publications were identified through targeted mechanistic searches. ClinicalTrials.gov was also searched through September 2026 using the terms “ketones AND sleep” to identify ongoing or recently completed clinical studies with sleep-related outcomes. We included mechanistic and animal studies when they measured sleep, sleep-related electroencephalographic (EEG) phenotypes, or highly sleep-relevant pathways, such as vesicular glutamate transport, ATP-sensitive potassium (KATP) channels, adenosine signaling, inflammasome biology, histone deacetylase (HDAC) inhibition, histone beta-hydroxybutyrylation, and circadian rhythms. Emphasis was placed on studies that directly measured sleep architecture, sleep EEG, sleep questionnaires, sleep-disordered breathing, or sleep-adjacent neurophysiology, while mechanistic papers were included when they plausibly linked ketone metabolism to sleep-regulatory pathways.

Human studies were included when they involved ketogenic diets, very-low-carbohydrate or very-low-calorie ketogenic diets, fasting/starvation physiology, exogenous ketones, or clinical ketogenic interventions and reported objective sleep architecture, subjective sleep quality, daytime sleepiness, sleep-disordered breathing, or sleep-related quality-of-life endpoints. Studies using polysomnography (PSG), ambulatory EEG, or EEG spectral analysis were treated as higher-inference sleep-architecture evidence than questionnaire-only studies. Interventions that combine ketosis with weight loss, caloric restriction, seizure control, migraine improvement, exercise, or respiratory changes were interpreted as multifactorial unless ketone exposure was experimentally isolated. Duplicate reports, studies without a relevant ketone or ketogenic exposure or a sleep-relevant mechanistic connection, and studies focused solely on ketoacidosis were not included, except when relevant to clinical safety. Because this was a narrative rather than systematic review, the search and selection strategy was designed to identify mechanistically and clinically informative literature rather than to support formal meta-analysis or quantitative evidence synthesis, and detailed exclusion counts were not prospectively maintained. The final review cited 89 peer-reviewed publications and two ongoing clinical-trial records. A simplified overview of the literature identification and selection process is provided in Supplementary Figure S1.

## 3. Ketone Biology and Sleep-Relevant Metabolic Context

### 3.1. BHB and AcAc Are Both Fuels and Signaling Molecules

β-Hydroxybutyrate (BHB) and acetoacetate (AcAc) are the principal circulating ketone bodies produced during hepatic fatty acid oxidation when carbohydrate availability is reduced, such as during fasting, carbohydrate restriction, prolonged exercise, or ketogenic dietary therapy. Although often discussed together as “ketones,” BHB and AcAc should not be treated as interchangeable metabolites. BHB and AcAc are interconverted by mitochondrial β-hydroxybutyrate dehydrogenase 1 (BDH1) in an NAD^+^/NADH-coupled near-equilibrium reaction; consequently, their relative abundance is influenced by the mitochondrial redox state [4,5]. This redox-dependent interconversion contributes to circulating BHB/AcAc proportions, although blood concentrations also reflect production, tissue uptake, oxidation, and clearance. AcAc can also undergo spontaneous decarboxylation to acetone, which is less studied in relation to sleep physiology.

Both BHB and AcAc cross the blood–brain barrier through monocarboxylate transporters and can be oxidized by neurons and glia, providing an alternative substrate for mitochondrial energy production when glucose availability or utilization is reduced. In this sense, ketone bodies are relevant to sleep because sleep is accompanied by state-dependent changes in substrate use, brain energetics, and cellular maintenance demands. However, their relevance is not limited to fuel provision. Ketone bodies also participate in signaling pathways that influence redox balance, neuronal excitability, and adaptive responses to energetic stress.

Distinguishing BHB from AcAc is especially important in sleep research. The most direct preclinical sleep-homeostasis experiment identified AcAc, rather than BHB, as the ketone species that increased slow-wave activity (delta power 0.5–4 Hz) during NREM sleep after central administration in mice [6]. This finding suggests that ketone effects on sleep may depend on the specific ketone species, route and timing of exposure, and brain-state context. It also cautions against using circulating BHB alone as a complete proxy for ketone signaling in sleep studies.

BHB has a broader body of signaling literature outside direct sleep measurement. Experimental studies link BHB to reduced oxidative-stress markers, including 4-hydroxynonenal, and to redox-sensitive gene programs [7]. BHB also regulates inflammatory and chromatin-associated pathways, including NLRP3 inflammasome activation, histone deacetylase activity, and histone β-hydroxybutyrylation [7,8,9]. These mechanisms are discussed in greater detail below because they may influence sleep indirectly through inflammation, oxidative stress resistance, circadian transcription, and cellular adaptation. At this stage, however, the key point is that BHB and AcAc represent overlapping but biologically distinct metabolic signals. Their effects on sleep architecture and sleep-dependent recovery are likely to depend not only on the presence of ketosis but also on ketone species, redox state, tissue utilization, and temporal pattern of exposure.

### 3.2. Brain Energetics During Sleep

Sleep is not a period of metabolic shutdown; it is a state-dependent reorganization of brain energy use. Across wakefulness, NREM sleep, and REM sleep, the brain changes neuronal firing patterns, neuromodulatory tone, synaptic activity, vascular dynamics, substrate handling, and cellular maintenance priorities. Thus, the key metabolic feature of sleep is not simply whether total energy expenditure is higher or lower than wakefulness, but how energy demand is redistributed across sleep stages, cell types, brain regions, and biochemical pathways.

NREM sleep is generally associated with reduced global energy demand relative to wakefulness, particularly during slow-wave sleep. Cortical and thalamocortical networks enter synchronized slow oscillations, sensory processing is reduced, sympathetic output generally declines, and whole-body energy expenditure often falls. However, this reduction in outward behavioral and sensory activity is accompanied by active maintenance processes. These include restoration of ionic gradients, synaptic recalibration, protein and membrane turnover, antioxidant defense, glycogen replenishment, and interstitial solute clearance. Therefore, NREM sleep is best understood as a metabolically reallocated state: some energy-consuming functions are downregulated, while restorative and homeostatic processes are prioritized.

REM sleep is metabolically distinct from NREM sleep and should not be treated as simply “lighter” or less important sleep. REM sleep combines peripheral muscle atonia with wake-like or regionally heightened neural activation, autonomic variability, and active processing in selected cortical, limbic, and brainstem networks. Functional imaging studies show that regional brain activity differs markedly between NREM and REM sleep, with REM sleep engaging neural systems more closely resembling wakefulness in some regions while suppressing others. This stage-specific physiology is important for ketone–sleep research because a metabolic intervention could plausibly increase NREM slow-wave activity, preserve REM sleep after physiological stress, or alter REM timing without producing a uniform increase in total sleep time.

Sleep-state energetics also involve changes in substrate metabolism. Aalling, Nedergaard, and DiNuzzo emphasized that glucose utilization, lactate dynamics, glycogen handling, and alternative substrate oxidation vary across the sleep–wake cycle, and that fatty-acid-derived substrates and ketone bodies may partly substitute for glucose as cerebral energy sources during sleep [1]. Lactate has emerged as a brain-state-sensitive metabolite, reflecting shifts in glycolysis, astrocyte–neuron interactions, and synaptic activity. Brain glycogen, stored primarily in astrocytes, is thought to decline during wakefulness and recover during sleep, supporting the idea that sleep contributes to restoration of local energy reserves [10]. Sleep is also associated with changes in extracellular space and interstitial solute clearance, including increased clearance of amyloid-β in sleeping mice, linking sleep-state physiology to metabolic waste handling [11].

This metabolic architecture provides a plausible rationale for why ketone bodies might interact with sleep physiology. Ketones can serve as oxidative substrates when glucose availability or utilization is reduced, and they also influence redox state, neuronal excitability, inflammation, and gene regulation. Their relevance may be greatest in contexts where sleep and metabolism are already under stress, including prolonged wakefulness, sleep deprivation, aging, neurological disease, impaired glucose metabolism, metabolic syndrome, neurodegeneration, and recovery from strenuous exercise. In these settings, ketones may alter the metabolic conditions under which sleep and wake states are generated, rather than acting as conventional hypnotics.

Aging and neurodegenerative risk are especially relevant because both sleep architecture and brain metabolism change with age. Older adults commonly show reduced slow-wave sleep, altered REM/NREM organization, greater sleep fragmentation, impaired metabolic flexibility, and increased vulnerability to oxidative and inflammatory stress. Alzheimer’s disease and related neurodegenerative conditions are also characterized by impaired cerebral glucose metabolism and disrupted sleep–wake regulation. Under these conditions, ketone availability could theoretically support neural function by providing an alternative substrate or by modifying stress-response pathways. However, this remains a hypothesis that requires direct testing with sleep physiology endpoints.

The relationship between sleep and ketone metabolism is also bidirectional. Ketosis may influence sleep-relevant pathways, but sleep loss, fasting, circadian phase, feeding timing, and energetic stress can also change ketone production and the AcAc/BHB ratio. Sleep deprivation increases sleep pressure and alters brain metabolism; fasting and time-restricted feeding alter ketogenesis and circadian metabolic rhythms; and exercise or caloric restriction can raise ketones while simultaneously changing catecholamines, appetite hormones, body temperature, and inflammatory tone. This bidirectionality is one reason human dietary studies are difficult to interpret unless energy intake, body weight, circadian timing, sleep opportunity, respiratory physiology, and measured ketone exposure are carefully controlled.

Therefore, brain energetics during sleep provide biological plausibility for ketone–sleep interactions but not proof that ketosis improves sleep. State-dependent substrate handling explains why ketones could matter, particularly under metabolic stress or impaired glucose utilization. It does not establish that diet-induced ketosis, exogenous BHB, consumption, or ketogenic therapy will reliably improve sleep architecture in healthy individuals. This distinction is essential: ketones may be sleep-relevant metabolic signals without being universal sleep-promoting agents.

## 4. Mechanistic and Preclinical Evidence

### 4.1. AcAc, Excitability, Adenosine, and Sleep Homeostasis

The strongest direct evidence that ketones influence sleep is the mouse study by Chikahisa et al. [6]. Six hours of sleep deprivation increased circulating ketones and altered the AcAc/BHB ratio. Central AcAc increased NREM SWA, whereas BHB did not produce the same effect, and AcAc was linked with reduced glutamate release. This supports AcAc as a potential sleep-pressure-related metabolic signal rather than only a passive fuel. Several mechanistic studies provide plausible substrates for this effect. AcAc can reduce vesicular glutamate transport by acting as a competitive antagonist to chloride. It also decreases quantal glutamate release and data suggest that it reduces the amount of glutamate per synaptic vesicle [12]. Glutamatergic signaling varies across the sleep–wake cycle, and greater glutamate exposure during wakefulness has been associated with increased sleep pressure [13]. Physiological ketone concentrations can also reduce central neuronal firing through KATP-channel (ATP-sensitive potassium channel) opening [14]. KATP-channels couple cellular metabolic state to membrane excitability and have been implicated in transitions between sleep and wake states [15].

Adenosine and cellular energetics provide an additional potential interface between ketone metabolism, neuronal excitability, and sleep homeostasis. Adenosine is closely coupled to cellular energy metabolism and contributes to homeostatic sleep pressure; extracellular adenosine generally increases during prolonged wakefulness and acts through A1 and A2A receptors to modulate sleep–wake circuitry [16,17,18]. Preclinical ketogenic studies further suggest that changes in energy metabolism can engage adenosine-dependent inhibitory signaling. Ketogenic feeding reduced adenosine kinase expression, and intact adenosine A1-receptor signaling was required for ketogenic-diet-mediated seizure suppression in mice [19]. In hippocampal slices, reduced extracellular glucose in the setting of sufficient intracellular ATP promoted ATP release and extracellular conversion to adenosine, followed by A1-receptor activation, KATP-channel opening, neuronal hyperpolarization, and reduced excitability [20].

Ketogenic metabolism can also increase mitochondrial capacity and cerebral energy reserves [21], suggesting that ketone-supported energetics may alter the relationship among ATP availability, purinergic signaling, and neuronal excitability rather than simply increasing neuronal activity. These mechanisms are directionally compatible with lower arousal drive or greater sleep stability, but their net effect depends on circuit location and vigilance state. Whether ketone-supported changes in ATP availability and adenosine signaling occur within sleep-regulatory circuits, influence the accumulation or resolution of homeostatic sleep pressure, or differ across brain regions, age, and disease states remains largely unknown.

### 4.2. Diet, Fasting, and Aging Animal Models

Dietary and fasting studies provide an important bridge between direct mechanistic experiments and human ketogenic interventions because they test ketone biology in the context of whole-organism physiology. However, these models also require careful interpretation. Fasting, caloric restriction, and ketogenic diets do not alter ketone availability in isolation; they also change glucose, insulin, leptin, ghrelin, corticosterone, fatty acid oxidation, body temperature, locomotor activity, feeding rhythms, and circadian entrainment. Sleep is highly sensitive to all of these variables. Thus, animal studies of diet and fasting are best understood as models of energetic state and metabolic adaptation rather than as pure tests of ketone bodies as sleep-promoting or wake-promoting agents.

Fasting models are particularly informative because they reveal how sleep–wake regulation adapts to reduced nutrient availability. In naturalistic terms, fasting creates competing physiological priorities: the organism may need to conserve energy through increased sleep or reduced activity, but it may also need to maintain wakefulness to forage. The direction of the sleep response therefore depends on species, circadian phase, adiposity, baseline metabolic reserve, and the neuroendocrine response to food deprivation. Danguir and Nicolaidis demonstrated this principle in rats by showing that starvation altered slow-wave sleep in a manner that depended on metabolic phenotype, with animals possessing greater fat stores showing less adverse sleep disruption during nutrient deprivation [22]. This finding is important because it implies that sleep responses to fasting are shaped by energy reserve and substrate availability, not simply by the absence of food.

Kondo et al. further positioned lipid and ketone metabolism within the adaptive sleep–wake response to fasting by studying peroxisome proliferator-activated receptor-α (PPARα) knockout mice [23]. PPARα is a nuclear receptor that coordinates hepatic fatty acid oxidation and ketogenesis during fasting. In wild-type animals, food deprivation engages lipid oxidation and ketone production as part of the metabolic response to reduced carbohydrate availability. In PPARα knockout mice, this fasting-induced ketone response is blunted, and the animals show impaired maintenance of wakefulness late in the dark phase [23]. This is a critical observation because it suggests that the capacity to mobilize lipid-derived fuels may support adaptive wakefulness during energetic challenge. In this context, ketone metabolism appears less like a simple hypnotic signal and more like part of a metabolic flexibility program that helps animals sustain appropriate vigilance when food is unavailable.

Together, the Danguir and Kondo studies [22,23] show that fasting-induced changes in sleep cannot be interpreted as direct ketone effects alone. Starvation increases ketones, but it also activates endocrine stress pathways, changes thermoregulation, alters locomotor drive, and shifts the balance between energy conservation and food-seeking behavior. These studies therefore place ketones within a broader energy-availability framework. They support the idea that lipid and ketone metabolism participate in vigilance-state regulation, but they do not establish that ketones alone are uniformly sleep-promoting or wake-promoting.

Dietary ketogenic models provide a complementary approach because they can raise ketone availability without complete food deprivation. These studies are particularly useful for understanding how sustained ketogenic metabolism affects baseline sleep architecture. Westmark et al. reported that a ketogenic diet altered sleep architecture in both C57BL/6J wild-type mice and Fmr1 knockout mice, a model relevant to Fragile X syndrome [24]. In that study, a ketogenic diet increased NREM sleep during the dark cycle and decreased wake bouts [24]. Because mice are nocturnal, the dark cycle represents the active phase, when wakefulness and feeding behavior are typically highest. Therefore, an increase in NREM sleep during the dark cycle may reflect reduced active-phase arousal, improved sleep consolidation, altered energy allocation, or a shift in the timing of sleep rather than a global increase in sleep across the entire day.

A related study of adult-onset ketogenic diet therapy similarly found increased sleep during the dark cycle in C57BL/6J wild-type and Fragile X model mice [25]. This replication is notable because it suggests that ketogenic diet effects on sleep are not limited to early-life dietary exposure and may emerge even when ketogenic therapy begins in adulthood. The use of Fmr1 knockout mice is also relevant because neurodevelopmental models often exhibit altered excitability, arousal regulation, and sleep architecture. In such models, a ketogenic diet may affect sleep partly by stabilizing neuronal excitability or reducing excessive wake fragmentation. However, the degree to which these findings generalize to healthy animals, aged animals, or humans with insomnia or metabolic disease remains uncertain.

Not all ketogenic diets produce the same sleep-stage effects. Zhuang et al. reported that a ketogenic diet containing medium-chain triacylglycerols reduced REM sleep duration in mice without producing major circadian disruption [26]. This finding contrasts with studies showing increased NREM sleep during the dark cycle [24,25] and with aged-rat findings showing REM enhancement in females after ketogenic interventions [27]. These differences likely reflect more than experimental noise. Ketogenic diets vary substantially in fat source, fatty acid chain length, protein content, carbohydrate restriction, caloric density, palatability, micronutrient composition, and achieved ketone profile. Medium-chain triglycerides are more rapidly absorbed and oxidized than many long-chain fats and may produce different patterns of ketone generation, gastrointestinal signaling, thermogenesis, and hepatic metabolism. Thus, the sleep effects of a ketogenic diet may depend not only on whether the diet is “ketogenic,” but on the specific formulation and the temporal pattern of metabolic signaling it produces.

The discrepant REM findings across dietary models are especially important. REM sleep is metabolically and neurophysiologically distinct from NREM sleep and may be particularly sensitive to age, sex, stress, thermoregulation, cholinergic tone, monoaminergic suppression, and metabolic state. A reduction in REM sleep after a medium-chain triglyceride ketogenic diet [26] should therefore not be viewed as contradicting REM enhancement in aged females after ketogenic interventions [27]. Rather, these findings suggest that REM sleep may be differentially regulated depending on diet formulation, species, age, biological sex, genotype, and baseline neural vulnerability. This reinforces the need to avoid treating “ketogenic diet” as a single standardized intervention in sleep research.

The recently published aged-rat study by Lark et al. adds an important aging and sex-difference dimension to this literature [27]. In 22–25-month-old Fischer-344 rats, both ketone ester gavage and a two-month ketogenic diet enhanced REM sleep in females, whereas ketone ester decreased REM sleep in males during the light phase [27]. These sex-specific responses are notable because aging is associated with altered sleep architecture, impaired metabolic flexibility, mitochondrial dysfunction, neuroinflammation, oxidative stress, and reduced resilience to physiological stressors. The finding that aged females showed REM enhancement after ketogenic interventions suggests that ketone availability may interact with sex-specific aging biology to influence sleep-state regulation.

Lark et al. also linked sleep outcomes with cognitive and molecular endpoints. A ketogenic diet improved novel place recognition in both females and males, suggesting that sustained ketogenic therapy may support memory-related outcomes in aged animals [27]. In females, a ketogenic diet also reduced hippocampal lipid peroxidation and microglial TREM2 signal, without broad suppression of NLRP3 [27]. These findings are important for two reasons. First, they suggest that sleep-stage changes, cognition, and oxidative/inflammatory markers may be linked in aged animals, although causality remains to be tested. Second, they indicate that ketogenic interventions may influence neurobiological aging through selective redox and microglial pathways rather than through uniform anti-inflammatory suppression.

The aged-rat data also highlight the importance of distinguishing acute exogenous ketone exposure from chronic ketogenic dietary adaptation. Ketone ester gavage can raise circulating ketones acutely without recreating all features of a ketogenic diet, whereas a chronic ketogenic diet alters macronutrient intake, substrate utilization, body composition, mitochondrial adaptation, inflammatory tone, and potentially gut–brain signaling. The observation that both interventions affected REM sleep in aged females suggests that ketone availability itself may be relevant, but differences between ketone ester and diet responses also indicate that the broader adaptive state matters. Future studies that measure nocturnal BHB and AcAc dynamics, brain ketone utilization, sex hormones, mitochondrial function, and EEG spectral features will be needed to determine whether REM changes reflect altered sleep regulation, improved metabolic sufficiency, or compensation for age-related neural vulnerability.

Overall, fasting, dietary, and aging models suggest that ketone biology is embedded in a broader network of energy availability, circadian timing, neural excitability, redox state, and sex-specific physiology. The animal literature does not support a single directional conclusion that ketones universally deepen sleep, increase REM sleep, or promote wakefulness. Instead, it supports a more conditional model: lipid and ketone metabolism can modify vigilance-state regulation, but the direction and magnitude of the effect depend on metabolic context, diet formulation, circadian phase, genotype, age, and sex. This framework is essential for translating preclinical findings to human studies, where ketogenic interventions often occur in the setting of obesity, diabetes, neurological disease, aging, exercise stress, sleep apnea, medication use, or caloric restriction.

### 4.3. Inflammatory and Epigenetic Pathways

Beyond their role as oxidative substrates, ketone bodies may influence sleep-relevant physiology through inflammatory and epigenetic signaling pathways. These mechanisms are especially relevant because sleep is tightly coupled to immune tone, oxidative stress, cellular repair, and transcriptional regulation. However, most evidence linking BHB to inflammation and epigenetic remodeling comes from experimental models that did not directly measure sleep. Therefore, these pathways should be interpreted as biologically plausible mechanisms through which ketones may indirectly modify sleep architecture, sleep continuity, or sleep-dependent recovery, rather than as established sleep-promoting effects.

BHB suppresses activation of the NLRP3 inflammasome and reduces downstream IL-1β and IL-18 signaling in experimental models [9]. This is relevant to sleep because inflammatory cytokines can influence sleep duration, NREM intensity, sleep fragmentation, and fatigue-like behavior. In inflammatory or metabolically stressed phenotypes, reduced inflammasome activation could plausibly improve sleep continuity or reduce inflammation-associated sleep disruption. However, cytokine effects on sleep are complex and depend on dose, timing, chronicity, brain region, and disease context. Acute immune signaling may promote aspects of NREM sleep, whereas chronic or excessive inflammatory signaling can fragment sleep, impair sleep quality, and alter circadian organization. Thus, the anti-inflammatory actions of BHB may be most relevant in states of pathological inflammation rather than in healthy individuals with intact sleep regulation.

BHB also has epigenetic actions that may influence sleep biology over slower timescales. Shimazu et al. demonstrated that BHB functions as an endogenous inhibitor of class I histone deacetylases, increasing histone acetylation and inducing transcriptional programs associated with oxidative stress resistance [7]. This mechanism provides a link between metabolic state and gene expression, suggesting that ketosis may modify cellular resilience during fasting, energetic stress, or inflammatory challenge. In the context of sleep, HDAC inhibition by BHB could theoretically support processes relevant to neural homeostasis, including antioxidant defense, mitochondrial adaptation, synaptic plasticity, and stress-response pathways. These effects would not necessarily produce an acute hypnotic response, but they may alter the cellular environment in which sleep-dependent repair and plasticity occur.

A second epigenetic mechanism is histone lysine β-hydroxybutyrylation. Xie et al. showed that elevated BHB can serve as a substrate for histone β-hydroxybutyrylation, a chromatin modification associated with starvation-responsive gene expression [8]. This finding expands the role of BHB from fuel and signaling metabolite to a direct contributor to metabolically sensitive chromatin remodeling. Because sleep and circadian physiology are both regulated by transcriptional timing, BHB-dependent chromatin modifications may provide a mechanism by which nutritional state influences longer-term sleep-relevant pathways, including metabolic stress resistance, neuroplasticity, inflammation, and circadian gene networks.

Together, these inflammatory and epigenetic pathways suggest that BHB may shape the biological context in which sleep occurs. Rather than acting as a conventional sedative, BHB may modulate sleep indirectly by reducing pathological inflammatory signaling, enhancing oxidative stress resistance, and altering gene-expression programs involved in cellular adaptation. These mechanisms may be particularly important in populations characterized by metabolic dysfunction, aging, neuroinflammation, obesity, diabetes, neurological disease, or sleep disruption associated with systemic inflammation. Future studies should test whether BHB-linked reductions in inflammasome activity, HDAC inhibition, or histone β-hydroxybutyrylation are associated with measurable changes in sleep architecture, EEG slow-wave activity, REM sleep, sleep fragmentation, or next-day cognitive and metabolic recovery.

### 4.4. Circadian Coupling of Ketone Metabolism and Sleep

Circadian regulation is a critical but often undermeasured dimension of ketone–sleep biology. Ketone bodies are not static metabolic substrates; their production, tissue utilization, and signaling effects vary across feeding–fasting cycles, behavioral state, and time of day [28,29]. This temporal organization is particularly relevant to sleep because the same ketone exposure may have different physiological effects depending on whether it occurs during the biological day, biological night, postprandial period, post-exercise recovery, prolonged fasting, or recovery from sleep deprivation.

The liver is the major source of circulating ketone bodies, and hepatic ketogenesis is tightly coordinated with circadian and nutrient-sensing pathways. Caloric restriction and time-restricted feeding generate distinct rhythms in circulating BHB, and functional molecular clocks are required for normal diet-induced ketone rhythmicity [29]. In mice, this clock-gated regulation involves interaction between the circadian transcriptional machinery and the peroxisome proliferator-activated receptor-α (PPARα) network, which regulates fatty acid oxidation and ketogenic enzyme expression [29]. These findings indicate that ketone bodies are not merely passive markers of energy deficit, but rhythmic outputs of coordinated circadian–metabolic regulation.

Ketogenic diets may also feed back on circadian organization. Preclinical studies show that ketogenic diets can reprogram tissue clocks and induce rhythmic oscillations of serum and intestinal BHB [30]. This suggests a bidirectional relationship in which the circadian clock gates ketone production, while ketogenic metabolism can modify peripheral clock function and tissue-specific transcriptional rhythms. Emerging data further suggest that BHB supplementation may partially restore disrupted circadian gene expression in metabolic tissues under high-fat diet conditions, potentially through AMPK activation and suppression of mTOR and NF-κB signaling [31]. These effects appear to be tissue-specific, with more prominent effects reported in adipose tissue than liver, reinforcing the idea that ketone signaling is temporally and anatomically context-dependent [31].

This circadian framework has important implications for sleep studies. First, the timing of ketone exposure should be considered an active experimental variable rather than a technical detail. A morning ketogenic signal, a pre-sleep ketone ester, and an overnight fasting-induced rise in ketones may engage different neuroendocrine, autonomic, and transcriptional states. Second, studies that measure only a single fasting morning BHB value may miss the ketone dynamics most relevant to sleep architecture, nocturnal brain metabolism, or next-day recovery. Third, interventions such as caloric restriction, time-restricted feeding, ketogenic diets, and exogenous ketone supplements may have different sleep effects because they impose different temporal patterns of ketone availability, insulin/glucose exposure, fatty acid oxidation, and peripheral clock entrainment [28,29,30].

More broadly, ketone rhythms exist within the larger chrononutrition network linking feeding time, fasting duration, peripheral clocks, hepatic metabolism, glucocorticoid signaling, and sleep–wake behavior. Feeding patterns are potent synchronizers of peripheral metabolic clocks, and the timing of food intake influences hepatic transcription through both clock-dependent and feeding-driven mechanisms [32]. Caloric restriction can also reprogram circadian clocks at transcriptional and post-transcriptional levels [33], while the glucocorticoid receptor contributes to the hepatic metabolic response to caloric restriction, including pathways that support ketone body production [34]. Lifespan and metabolic benefits of caloric restriction in mice are enhanced when feeding is aligned with the active phase and includes a prolonged daily fasting interval, conditions that also promote rhythmic ketone production [35]. Although these studies were not designed primarily to measure sleep, they support the concept that ketone biology is embedded within a broader temporal architecture of energy availability.

Therefore, ketones and circadian rhythms should be considered part of a bidirectional regulatory loop: circadian clocks shape when ketones are produced, and ketones may in turn influence nutrient-sensing and clock-related pathways [28,29,31]. For ketone–sleep research, this means that future studies should measure the timing, magnitude, and duration of nocturnal ketone exposure; distinguish diet-induced from supplement-induced ketone rhythms; and incorporate circadian phase markers when possible. This approach will be essential for determining whether ketones influence sleep architecture directly, indirectly through peripheral metabolic entrainment, or conditionally depending on circadian alignment. The key mechanistic and preclinical studies discussed in this section are summarized in Table 1.

## 5. Human Evidence and Clinical Relevance

Human evidence linking ketones to sleep spans several distinct clinical and experimental contexts, including ketogenic dietary therapy for epilepsy, acute carbohydrate restriction, ketogenic interventions in metabolic and neurological populations, sleep-disordered breathing, and exogenous ketone supplementation. These studies should not be interpreted as testing a single intervention. Nutritional ketosis, therapeutic ketogenic diets, fasting, very-low-calorie ketogenic diets, and exogenous ketone ingestion differ in their effects on ketone species, glucose and insulin dynamics, weight loss, fatty acid oxidation, circadian feeding cues, respiratory physiology, medication exposure, and disease activity. Therefore, the human literature is best organized by clinical context and intervention type rather than by the presence or absence of ketosis alone.

### 5.1. Epilepsy: The Foundational Human Model for Ketogenic Metabolic Therapy

Epilepsy is the most established human context for ketogenic metabolic therapy and should be considered a central translational model for ketone–sleep research. Ketogenic dietary therapies are used clinically for drug-resistant epilepsy, where the primary therapeutic goal is not sleep induction but stabilization of pathological neural excitability and a reduction in seizure burden. This distinction is important because sleep may improve during ketogenic therapy through several interacting pathways: direct effects of ketone metabolism on sleep-regulatory mechanisms, reduced seizure frequency, decreased nocturnal epileptiform activity, improved daytime alertness, medication-related changes, or broader improvements in cognition, behavior, and quality of life.

The relevance of epilepsy to ketone–sleep biology is strengthened by the bidirectional relationship between sleep and epileptiform activity. Sleep deprivation and poor sleep quality can increase seizure susceptibility, while seizures, interictal epileptiform discharges, antiseizure medications, and epilepsy-related neurodevelopmental dysfunction can disrupt sleep continuity, sleep architecture, daytime vigilance, and behavior. Therefore, ketogenic therapy in epilepsy provides a clinically meaningful setting in which metabolic modulation of brain excitability may influence both sleep and wake states.

Pasca et al. reviewed the effects of ketogenic dietary therapies on sleep in neurological populations and found that the literature is dominated by epilepsy studies, with 18 of 20 included studies involving epilepsy as the primary diagnosis [36]. Across these studies, reported sleep-related outcomes included improved sleep quality, fewer awakenings, quieter nights, reduced daytime sleepiness, better morning awakening, decreased sleep anxiety, increased REM sleep, and reductions in interictal epileptiform discharges during sleep [36]. However, the review also emphasized substantial heterogeneity in study design, sleep measurement, diet type, timing of assessment, age range, and whether sleep outcomes were measured independently from seizure outcomes [36]. Thus, the epilepsy literature provides the richest human ketogenic therapy context for sleep but also illustrates the major challenge of separating ketosis-specific effects from seizure reduction and broader clinical improvement.

The pediatric epilepsy literature includes some of the most direct sleep-architecture evidence for ketogenic dietary therapy. Hallböök, Lundgren, and Rosén evaluated children with therapy-resistant epilepsy using ambulatory polysomnography before and after ketogenic diet initiation [37]. After three months of ketogenic therapy, total sleep and total night sleep decreased, REM sleep increased, and NREM stage 2 sleep decreased, while slow-wave sleep and stage 1 sleep were unchanged. At follow-up, increased REM sleep was associated with improved quality of life, and daytime sleep was reduced at 12 months [37]. These findings are important because they suggest that ketogenic therapy can alter objective sleep architecture in children with epilepsy, but the pattern is not a simple global increase in sleep. Rather, ketogenic therapy appeared to reorganize the sleep and wake distribution, with changes in REM sleep, daytime sleep, and quality-of-life measures. Whether these sleep responses differ from those in adults at comparable ketone exposure is unknown and represents an important developmental question.

A related study from the same group examined epileptiform activity and found that a ketogenic diet reduced interictal epileptiform discharges during sleep in children with therapy-resistant epilepsy [38]. Pasca et al. note that reductions in interictal discharges were observed across sleep stages, including NREM stage 2, slow-wave sleep, and REM sleep, and were closely related to seizure-frequency reduction [36]. This finding is particularly relevant to the ketone–sleep model because interictal epileptiform discharges during sleep can disrupt sleep continuity and interfere with sleep-dependent cognition. Reduced nocturnal epileptiform activity may therefore improve sleep quality indirectly by stabilizing neural activity during sleep, even if the primary therapeutic mechanism is anticonvulsant rather than hypnotic.

Several additional EEG studies support the importance of nocturnal epileptiform activity as a sleep-relevant outcome during ketogenic therapy. Ebus et al. prospectively studied children and adults with refractory epilepsy and reported that early 24-h EEG changes, including reductions in nocturnal interictal epileptiform discharges, were associated with clinical response to ketogenic diet therapy [39]. Wu et al. similarly reported that a ketogenic diet reduced the epileptiform discharge index during sleep in children with pharmacoresistant epileptic encephalopathy, with early sleep-EEG improvement related to later seizure reduction [40]. Reyes et al. studied patients with epileptic encephalopathy with electrical status epilepticus during slow sleep and reported improvement in epileptiform abnormalities during sleep EEG in association with ketogenic diet treatment and seizure reduction [41]. Peng et al. also reported sleep improvement using EEG-based assessment in febrile infection-related epilepsy syndrome treated with a ketogenic diet [42]. Together, these studies suggest that sleep EEG may be a particularly informative endpoint for ketogenic therapy because it captures both sleep-state physiology and pathological network activity.

Subjective and caregiver-reported sleep improvements have also been reported across epilepsy cohorts, although these data are less specific than PSG or EEG outcomes. Parents and clinicians have described improved sleep quality, reduced awakenings, quieter nights, improved daytime sleepiness, better morning awakening, improved alertness, and improved daily activity during ketogenic therapy in selected populations [43,44,45,46]. These outcomes are clinically meaningful because they align with family-centered goals of epilepsy care, including improved daily function, behavior, endurance, self-care, and quality of life. However, they are also difficult to interpret mechanistically because many were collected through parent report or clinical observation rather than standardized sleep instruments or objective sleep recordings.

This epilepsy literature supports a broader model in which ketones may improve sleep by improving the quality and stability of wakefulness. In epilepsy, ketogenic therapy may reduce seizure burden and epileptiform activity, improve attention or behavior, and reduce pathological daytime sleepiness. These wake-state improvements may then alter subsequent sleep pressure, sleep continuity, REM/NREM organization, and perceived sleep quality. Hallböök et al. provide an example in which ketogenic therapy was associated with increased REM sleep, reduced daytime sleep, improved alertness, and improved quality of life [37,38]. The broader EEG literature further suggests that reduced pathological activity during sleep may improve the integrity of both nocturnal sleep and next-day function [39,40,41,42].

Therefore, epilepsy studies should not be treated as peripheral clinical examples in ketone–sleep research. They represent the human setting in which ketogenic metabolic therapy is most established, the mechanistic link between metabolism and neural excitability is strongest, and objective EEG endpoints are most developed. At the same time, epilepsy studies underscore the interpretive complexity of the field: when sleep improves during ketogenic therapy, the effect may reflect ketone signaling, seizure reduction, reduced nocturnal epileptiform burden, medication changes, improved wake-state quality, or their interaction. Future epilepsy studies of ketogenic therapy should therefore incorporate baseline and follow-up sleep assessment, PSG or sleep EEG when feasible, standardized sleep questionnaires, daytime alertness and cognition measures, seizure and interictal discharge quantification, and nocturnal ketone measurements.

### 5.2. Acute Carbohydrate Restriction and Early Sleep Architecture Studies

The earliest human evidence linking nutritional state, ketosis, and sleep architecture comes from fasting and macronutrient-manipulation studies conducted before modern continuous ketone profiling, wearable sleep assessment, or contemporary EEG spectral methods were available. These studies remain important because they provide some of the only controlled human sleep-architecture data suggesting that short-term shifts in energy availability and carbohydrate intake can alter NREM/REM organization. However, they should be interpreted as studies of acute metabolic perturbation rather than as definitive tests of ketone-specific sleep effects.

Complete starvation represents the most extreme physiological model of endogenous ketosis. In young men, several days of starvation were associated with increased slow-wave sleep and reduced REM sleep, with reversal after refeeding [47]. This pattern is consistent with the concept that acute energy deficit can shift sleep toward deeper NREM expression while suppressing REM sleep. However, starvation also changes many non-ketone variables, including glucose, insulin, leptin, ghrelin, cortisol, sympathetic tone, thermoregulation, body weight, hunger, and psychological stress. Therefore, the observed increase in slow-wave sleep cannot be attributed to ketones alone. Rather, starvation studies suggest that the sleeping brain is sensitive to systemic energy availability and may reorganize sleep architecture during sustained metabolic challenge.

Early controlled macronutrient studies extended this idea by testing whether sleep architecture changes under isocaloric dietary conditions. Phillips et al. studied eight healthy young men exposed to diets differing in carbohydrate and fat content [48]. Compared with a high-carbohydrate/low-fat diet, the low-carbohydrate/high-fat condition was associated with greater NREM sleep, supporting the idea that macronutrient composition can influence sleep structure even when total caloric intake is controlled [48]. This study is valuable because it partially separates macronutrient composition from caloric deprivation. Nevertheless, it did not include modern blood BHB or AcAc measurements, and the small sample size limits inference regarding sex differences, interindividual metabolic flexibility, or the degree to which observed sleep changes were mediated by ketosis versus carbohydrate availability, fat oxidation, insulin dynamics, or other diet-related signals.

Kwan et al. conducted a complementary short-term low-carbohydrate diet study in women [49]. During a one-week low-carbohydrate diet, participants showed increased plasma BHB and reduced glucose, confirming a measurable shift toward ketosis and lower carbohydrate availability [49]. Sleep assessment showed decreased REM latency but no major change in total sleep time or overall sleep-stage distribution [49]. These findings are important because they complicate a simple model in which low-carbohydrate intake uniformly increases slow-wave sleep or suppresses REM sleep. Instead, they suggest that ketosis and reduced glucose availability may alter specific features of REM regulation, such as REM timing, without necessarily producing broad changes in total sleep duration or stage composition over a one-week period. The inclusion of women is also notable, given that sex differences may influence both ketosis and sleep architecture, although the study was not designed to test sex-specific mechanisms.

Afaghi et al. later provided one of the most frequently cited modern controlled feeding studies of acute carbohydrate restriction and sleep architecture [50]. In healthy men, a 48-h very-low-carbohydrate diet increased the percentage of slow-wave sleep and reduced the percentage of REM sleep compared with a mixed diet [50]. This finding is consistent with the starvation literature in suggesting that acute carbohydrate restriction may favor deeper NREM sleep and suppress REM expression in some healthy adults. Because the intervention was short and controlled, the study is particularly useful for identifying acute sleep responses during the early transition toward carbohydrate restriction. However, it does not establish whether the observed effects were driven by BHB, AcAc, the AcAc/BHB ratio, reduced glucose and insulin, increased fatty acid oxidation, altered amino acid availability, or early ketogenic adaptation.

Taken together, these early studies support the broader conclusion that sleep architecture is sensitive to the acute nutritional and metabolic state. Across starvation, low-carbohydrate feeding, and very-low-carbohydrate dietary conditions, the most consistent signal is a potential shift toward increased slow-wave sleep or altered REM regulation in some healthy adults [47,48,49,50]. However, the evidence is not uniform, and the studies differ in sex, duration, dietary design, degree of ketosis, sleep methodology, and whether ketosis was measured directly. These limitations are important because acute carbohydrate restriction may not reflect chronic ketogenic adaptation, therapeutic ketogenic diet use, or exogenous ketone supplementation. Short-term carbohydrate restriction can also produce transitional symptoms, including hunger, sympathetic activation, gastrointestinal changes, electrolyte shifts, or sleep disturbance in some individuals, which may differ from longer-term responses after adaptation.

In contrast to the acute carbohydrate-restriction literature, a randomized crossover trial in healthy adults found no improvement in subjective sleep quality, mood, or morning vigilance after three weeks of nutritional ketosis [51]. This finding suggests that ketogenic effects on sleep may be modest or absent in healthy good sleepers, may attenuate after adaptation, or may not be detectable using subjective outcomes alone. It also reinforces the possibility that ketosis-related sleep effects may be most evident when objective sleep architecture is measured, when baseline sleep is impaired, or when ketosis occurs in the context of physiological stress, neurological disease, metabolic dysfunction, or recovery from high wake-state demand.

Therefore, these studies are best interpreted as evidence that acute macronutrient shifts and endogenous ketosis can be associated with changes in human sleep architecture, not as proof that ketones directly or universally deepen sleep. They provide an important historical and physiological foundation for ketone–sleep research, but future studies should repeat these paradigms using contemporary methods, including polysomnography with EEG spectral analysis, repeated nocturnal BHB and AcAc measurements, respiratory monitoring, standardized circadian timing, and adequate representation of women, older adults, and metabolically vulnerable populations.

### 5.3. Non-Epilepsy Neurological and Psychiatric Populations

Beyond epilepsy, ketogenic dietary therapies have been examined in several neurological and psychiatric populations in which sleep disturbance, fatigue, cognitive symptoms, pain, mood dysregulation, and altered daytime function commonly coexist. These studies are clinically relevant because they test ketogenic interventions in populations with impaired brain-state regulation, but they are also difficult to interpret mechanistically. In many cases, improved sleep may reflect changes in the underlying neurological or psychiatric condition, reduced pain or fatigue, improved mood, altered daytime alertness, or weight and metabolic changes rather than a direct effect of ketones on sleep-generating circuits.

One early example is narcolepsy, a disorder characterized by excessive daytime sleepiness, REM sleep dysregulation, and impaired wake-state stability. A small uncontrolled study of a low-carbohydrate ketogenic diet reported improvement in narcolepsy symptom scores [52]. Although limited by sample size, lack of a control group, and reliance on symptom-based outcomes, this study is conceptually important because narcolepsy is fundamentally a disorder of sleep–wake state instability. The findings raise the possibility that ketogenic metabolism may influence not only nocturnal sleep but also daytime vigilance and state-boundary control. However, objective measures such as polysomnography, multiple sleep latency testing, actigraphy, and nocturnal ketone profiling would be needed to determine whether ketogenic therapy modifies REM regulation, sleepiness, or wake stability directly.

More recent neurological cohorts have relied largely on questionnaires rather than objective sleep architecture. In multiple sclerosis, Merlino et al. reported improvements in Pittsburgh Sleep Quality Index (PSQI) and Epworth Sleepiness Scale (ESS) scores after six months of ketogenic diet therapy [53]. Perlman et al. similarly reported improved sleep-disorder screening profiles during a six-month ketogenic intervention in relapsing multiple sclerosis [54]. These findings are clinically meaningful because sleep disturbance and fatigue are major contributors to disability and quality of life in multiple sclerosis. However, interpretation is complicated by the fact that ketogenic dietary therapy may also affect fatigue, body weight, inflammation, mood, pain, and perceived neurological function. Thus, improved sleep scores in multiple sclerosis may reflect a combination of sleep-specific effects and broader improvements in disease-related symptom burden.

Migraine provides another important neurological context because sleep disturbance, circadian disruption, pain, and metabolic stress are closely linked to headache susceptibility. Several migraine cohorts have reported improved subjective sleep quality and reduced daytime sleepiness during ketogenic therapy [55,56,57]. These findings are plausible because reductions in migraine frequency, pain burden, photophobia, medication use, and fatigue could secondarily improve sleep continuity and perceived sleep quality. At the same time, they underscore a recurring challenge in the human literature: when sleep improves during ketogenic therapy for a neurological disorder, the improvement may be mediated by reduced disease activity rather than by a direct nocturnal effect of ketosis.

Ketogenic dietary interventions have also been explored in psychiatric populations, where sleep disruption is common and tightly linked to mood, cognition, circadian instability, and metabolic dysfunction. In psychiatric cohorts, ketogenic intervention has been associated with improved subjective sleep quality [58]. This observation is relevant because metabolic dysfunction, neuroinflammation, altered mitochondrial function, and circadian disruption are increasingly recognized as contributors to psychiatric illness. However, psychiatric sleep outcomes are particularly vulnerable to confounding by mood improvement, medication changes, weight loss, daytime routine, and expectancy effects. Future studies in psychiatric populations should therefore combine validated sleep questionnaires with actigraphy or PSG, mood and cognition measures, medication tracking, and objective metabolic biomarkers.

Sleep responses during ketogenic therapy may also be bidirectional, particularly during early dietary adaptation. A recent systematic analysis of ketogenic-diet adverse events identified sleep disturbance during adaptation and insomnia among reported psychiatric adverse events, although these events were not related to measured ketone concentrations [59]. Acute very-low-carbohydrate feeding has similarly been associated with increased arousals during lighter NREM sleep; notably, this effect was observed early in the intervention and was not sustained after 48 h [50]. Current human studies have shown varied relationships between circulating BHB concentration and sleep: sustained nutritional ketosis has produced neutral subjective sleep outcomes in healthy populations [51], while pre-sleep D-BHB and ketone ester interventions have produced favorable subjective or objective sleep outcomes in selected contexts [60,61]. More recently, ketogenic precursor supplementation during experimental sleep restriction increased wearable-derived deep sleep while also improving selected waking-performance measures [62] (NCT05519644). Thus, sleep disruption during ketogenic adaptation is clinically plausible, but whether it reflects ketone concentration itself versus changes in carbohydrate availability, energy balance, fasting, fluid/electrolyte status, or other aspects of metabolic adaptation remains unresolved.

Taken together, non-epilepsy neurological and psychiatric studies suggest that ketogenic interventions may improve patient-reported sleep and daytime sleepiness in selected populations, particularly when sleep disturbance is embedded within broader neurological, pain, fatigue, or mood syndromes. However, these data do not yet establish that ketones directly improve sleep architecture. A more conservative interpretation is that ketogenic therapy may improve the clinical conditions that disrupt sleep and wake quality, thereby supporting healthier 24-h sleep–wake organization. This interpretation is consistent with the broader model that ketones may improve subsequent sleep indirectly by improving the metabolic, inflammatory, cognitive, or neurological quality of wakefulness.

### 5.4. Metabolic Disease, Obesity, and Patient-Reported Sleep Quality

Metabolic disease and obesity are highly relevant to ketone–sleep research because insulin resistance, adiposity, systemic inflammation, glycemic variability, sleep fragmentation, fatigue, and sleep-disordered breathing often coexist. In these populations, ketogenic interventions may improve sleep through multiple pathways, including weight loss, improved glycemic control, reduced nocturia, lower inflammatory tone, improved energy stability, reduced reflux, altered appetite hormones, and changes in medication exposure. Therefore, improvements in patient-reported sleep during nutritional ketosis are clinically important but cannot be attributed to ketones alone without careful control conditions.

A one-year continuous care intervention using nutritional ketosis in adults with type 2 diabetes and prediabetes reported improved patient-reported sleep measured by the Pittsburgh Sleep Quality Index [63]. Participants entered the prediabetes/metabolic-syndrome cohort through the parent trial, whose eligibility criteria included a diagnosis of metabolic syndrome, BMI > 30 kg/m^2^, and HbA1c > 5.6%. Global PSQI scores improved after one year in both the type 2 diabetes and prediabetes nutritional ketosis groups. The proportion of poor sleepers also decreased, from 68.3% to 56.5% in the type 2 diabetes group and from 77.9% to 48.7% in the prediabetes group [63]. These sleep improvements occurred alongside broader metabolic improvements. In the prediabetes cohort, the continuous care intervention was associated with improvements in glycemic status, body weight, and other cardiometabolic risk factors, with a substantial proportion of participants achieving normoglycemia during follow-up. The T2D cohort likewise showed improvements in glycemic control, body weight, and medication requirements [64]. These findings are clinically meaningful because poor sleep is common in insulin resistance and diabetes and may worsen glycemic control, appetite regulation, cardiovascular risk, and daytime function.

However, this intervention was multifactorial. Participants experienced changes in weight, glycemia, medication exposure, dietary behavior, health coaching, and lifestyle routines [63]. Each of these factors could plausibly influence sleep quality. Improved glycemic control may reduce nocturia, nocturnal hypoglycemia, and glucose variability; weight loss may improve comfort and breathing; medication changes may alter sleepiness or nighttime awakenings; and structured lifestyle support may improve regularity and self-efficacy. Thus, the study supports the clinical relevance of nutritional ketosis for sleep quality in metabolic disease, but it does not isolate ketone bodies as the direct causal mediator.

Very-low-calorie ketogenic diets in obesity provide a related but distinct context because caloric restriction and weight loss are central components of the intervention. In women with overweight or obesity, a very-low-calorie ketogenic diet improved PSQI scores, and changes in sleep quality were related to changes in fat mass and inflammatory markers such as high-sensitivity C-reactive protein [65]. This finding suggests that improved sleep during ketogenic weight-loss interventions may be mediated partly by reductions in adiposity and systemic inflammation. Such mechanisms are clinically plausible because adipose tissue inflammation, pain, reflux, sleep-disordered breathing, and metabolic inflexibility can all contribute to poor sleep quality.

These metabolic and obesity studies are important for clinical translation because they reflect the populations most likely to use ketogenic dietary therapy in practice. However, they also illustrate why sleep outcomes in metabolic studies should be interpreted with caution. A ketogenic intervention may raise BHB, but it may simultaneously reduce body weight, insulin levels, glucose variability, inflammatory markers, blood pressure, and medication burden. The sleep benefit may arise from any combination of these changes. Future metabolic trials should therefore measure sleep prospectively using validated questionnaires, actigraphy, and ideally PSG in selected participants, while also tracking nocturnal ketone dynamics, weight loss, glycemic variability, medication changes, inflammatory markers, and sleep-disordered breathing.

### 5.5. Sleep-Disordered Breathing and Respiratory Physiology

Sleep-disordered breathing should be considered separately from general sleep quality because its mechanisms are distinct. In obstructive sleep apnea and obesity hypoventilation syndrome, sleep disruption is driven not only by sleep-stage regulation but also by upper-airway collapsibility, ventilatory control, carbon dioxide handling, oxygenation, arousal threshold, body composition, fluid shifts, and respiratory mechanics. Ketogenic interventions may influence these outcomes through weight loss, changes in respiratory exchange ratio, reduced carbon dioxide production, altered body water, changes in leptin and insulin, and improved ventilatory efficiency. These mechanisms are clinically important, but they do not necessarily indicate direct effects of ketones on sleep architecture.

Osman et al. reported that a ketogenic diet increased blood ketones from approximately 0.14 mmol/L to about 2 mmol/L and acutely improved gas exchange, sleep apnea severity, and nocturnal oxygenation in patients with obesity hypoventilation syndrome [66]. Rebound hypercapnia after return to a regular diet further supports the idea that macronutrient composition and substrate oxidation can influence ventilatory physiology [66]. This study is particularly relevant because it suggests that ketogenic metabolism may affect sleep-related breathing through respiratory and metabolic mechanisms, potentially independent of long-term weight loss. Lower respiratory exchange ratio and reduced carbon dioxide production could be especially relevant in hypercapnic phenotypes.

Earlier adolescent obesity data similarly suggest that ketogenic weight-loss interventions may improve sleep abnormalities during substantial metabolic and body-composition change [67]. However, in obesity-related sleep disruption, improvements may be driven by reduced fat mass, improved airway mechanics, reduced leptin/insulin resistance, reduced systemic inflammation, or improved nocturnal oxygenation rather than ketone signaling in the central nervous system. These studies should therefore be interpreted as evidence that ketogenic interventions can influence sleep-relevant respiratory physiology, not as evidence that ketosis directly improves NREM or REM regulation.

For clinicians, this distinction is essential. In patients with sleep apnea or obesity hypoventilation syndrome, ketogenic dietary therapy may be a useful metabolic intervention, but it should not replace evidence-based respiratory treatments such as CPAP, bilevel positive airway pressure, or other indicated therapies. Sleep-disordered breathing studies should include objective respiratory endpoints, including apnea–hypopnea index, oxygen saturation, carbon dioxide measures, arousal index, sleep-stage distribution, and body-composition data. Future trials should also distinguish acute substrate effects from longer-term weight-loss effects and should measure whether improvements in nocturnal breathing translate into improved daytime alertness, cognition, cardiometabolic risk, and sleep architecture.

### 5.6. Exogenous Ketones and Acute Physiological Stress

Exogenous ketone consumption provides a useful experimental approach for raising circulating ketone concentrations without requiring sustained carbohydrate restriction or long-term dietary adaptation. This distinction is important because exogenous ketosis can acutely increase circulating BHB while leaving many features of ketogenic dietary therapy unchanged, including habitual macronutrient intake, body weight, insulin sensitivity, gut microbiome composition, fatty acid oxidation, and circadian feeding patterns. Therefore, exogenous ketone studies are useful for testing acute ketone availability, but they should not be assumed to reproduce the full metabolic state induced by fasting or ketogenic diet therapy.

Different exogenous ketone preparations also produce distinct metabolic profiles. Ketone esters, ketone salts, and direct BHB beverages differ in pharmacokinetics, achieved D-BHB concentration, mineral load, acid–base effects, gastrointestinal tolerability, prandial sensitivity, and AcAc/BHB exposure. This distinction is particularly relevant to sleep because most clinical exogenous-ketone studies characterize exposure primarily by circulating BHB, whereas AcAc is rarely measured despite the direct preclinical evidence linking AcAc to NREM slow-wave activity. Because BHB and AcAc interconvert according to metabolic and redox state [4,5], the administered ketone formulation and measured BHB concentration may not fully represent the ketone species reaching or acting within the brain. These differences matter for sleep studies because a pre-sleep ketone intervention may influence not only ketone availability but also gastrointestinal tolerability, mineral and electrolyte exposure, acid–base balance, nocturnal glucose availability, and respiratory physiology, any of which could potentially affect sleep independently of central ketone signaling [68,69]. Thus, exogenous ketone formulation, dose, timing, and meal state should be treated as active experimental variables.

The strongest objective human evidence on exogenous ketones and sleep comes from Robberechts et al., who tested ketone ester ingestion in well-trained male cyclists after a strenuous exercise day that included late-evening high-intensity interval training ending one hour before sleep [61]. Participants received ketone ester after exercise and again 30 min before bedtime, producing D-BHB concentrations of approximately 3 mM during the early part of the night [61]. Strenuous exercise reduced REM sleep and increased wakefulness after sleep onset, whereas ketone ester ingestion improved sleep efficiency, counteracted the exercise-induced REM reduction, and reduced wakefulness after sleep onset [61]. This study is important because it suggests that exogenous ketosis may be most effective when sleep is challenged by a preceding period of high metabolic and physiological stress. These findings also support a broader model in which ketones may influence sleep by improving recovery from the preceding waking state. Exercise-induced sleep disruption reflects prior wake-state load, sympathetic activation, substrate depletion, thermal stress, catecholamine signaling, and recovery demand. The Robberechts study therefore suggests that ketosis, generated by consuming exogenous ketones, may influence sleep within a broader physiological recovery response rather than acting as a conventional hypnotic [61]. Therefore, this literature should be interpreted as supporting a recovery-state hypothesis rather than proving that exogenous ketones act as direct hypnotic agents.

Appetite and feeding-related mechanisms may also be relevant. Stubbs et al. reported that a ketone ester drink lowered ghrelin and appetite [70]. This finding is indirect with respect to sleep, but biologically relevant because hunger, late-night feeding, circadian meal timing, and appetite hormones can influence sleep onset, arousal, and nocturnal metabolic state. A pre-sleep ketone intervention that reduces hunger or modifies feeding-related signals could theoretically affect sleep timing or continuity, particularly during caloric restriction, evening hunger, or post-exercise energy deficit. However, this pathway remains speculative because appetite studies have generally not included polysomnography, nocturnal arousal measures, or sleep-stage outcomes.

The hypoxia literature provides an important counterpoint. In a related sleep study, ketone ester ingestion during simulated altitude or hypoxic sleep improved oxygenation only under more severe desaturation conditions but did not rescue hypoxia-induced disruption of sleep architecture [71]. This suggests that exogenous ketones may be insufficient when respiratory stressors dominate sleep physiology. It also reinforces the need to distinguish sleep-stage regulation from respiratory physiology. Ketone ester may influence oxygenation, substrate oxidation, or ventilatory responses under some conditions, yet these changes may not translate into improved REM/NREM architecture when hypoxic arousal, oxygen desaturation, or ventilatory instability are the primary drivers of sleep disruption.

Katsuya et al. tested a different approach: repeated pre-sleep D-BHB ingestion for 14 days in healthy Japanese adults using a randomized, double-blind, placebo-controlled design [60]. D-BHB improved several subjective sleep-quality domains, including sleepiness on rising and frequent dreaming in both D-BHB groups, with additional dose-specific effects on sleep maintenance and refreshment [60]. This study suggests that repeated pre-sleep D-BHB may affect perceived sleep quality. However, because the study relied on subjective sleep outcomes rather than polysomnography, actigraphy, or EEG spectral analysis, it remains unclear whether D-BHB changed sleep architecture, sleep continuity, REM/NREM organization, or morning perception of sleep without objective sleep-stage changes.

Sympathoadrenal responses also appear to vary substantially with metabolic and physiological context. In healthy men, three days of an isoenergetic diet containing ≤5% carbohydrate increased resting BHB to approximately 2.4 mmol/L together with higher resting noradrenaline and greater catecholamine responses to exercise [72]. However, this dietary paradigm cannot distinguish effects of ketosis from concurrent carbohydrate restriction, glycogen depletion, reduced glucose and insulin, or other counter-regulatory adaptations. During endurance-training overload, repeated ketone ester supplementation attenuated the training-associated increase in nocturnal adrenaline and noradrenaline excretion [73]. In the Robberechts et al. sleep study, pre-sleep ketone ester ingestion increased nocturnal urinary dopamine excretion without increasing adrenaline or noradrenaline and was accompanied by improved sleep efficiency [61]. Acute ketone monoester administration has also been shown not to alter directly measured muscle sympathetic nerve activity at rest or during laboratory stress [74]. Together, these studies illustrate context-dependent sympathoadrenal responses across carbohydrate restriction and ketosis induced by exogenous sources.

Overall, exogenous ketone studies support a conditional rather than universal sleep-promoting model. The strongest objective evidence comes from ketone ester ingestion after strenuous evening exercise, where exogenous ketosis improved sleep efficiency and preserved REM sleep during a metabolically stressful recovery condition [61]. In contrast, ketone ester did not rescue hypoxia-driven sleep-architecture disruption [71], and D-BHB beverage studies currently provide subjective but not objective sleep evidence [60]. These findings suggest that exogenous ketones may be most relevant when sleep is disrupted by energetic stress, exercise recovery, autonomic or metabolic stress, waking-state stressors, hunger, or altered substrate availability, but may be less effective when respiratory instability or hypoxia is the dominant driver.

Future exogenous-ketone sleep studies should directly compare ketone formulations, including ketone esters, ketone salts, direct D-BHB, and AcAc-generating approaches where feasible. Studies should measure nocturnal D-BHB, AcAc, glucose, insulin, free fatty acids, acid–base status, gastrointestinal tolerability, respiratory parameters, and circadian timing. Sleep outcomes should include polysomnography, EEG spectral analysis, REM/NREM architecture, wakefulness after sleep onset, arousal index, sleep spindles, subjective sleep quality, next-day alertness, cognition, and recovery markers. Such designs would help determine whether exogenous ketones affect sleep directly through brain-state mechanisms, indirectly through improved wake-state recovery, or conditionally depending on the physiological stressor preceding sleep.

### 5.7. Ketones, Wake Quality, and the 24-Hour Sleep–Wake Cycle

An important interpretation emerging from the human literature is that ketones may influence sleep indirectly by improving the metabolic quality, neural stability, or recovery state of wakefulness. Sleep architecture is shaped not only by processes that occur during the night but also by the physiological conditions established during the preceding waking period. If ketogenic metabolism improves daytime alertness, seizure control, cognitive efficiency, exercise recovery, inflammatory tone, autonomic balance, or metabolic resilience, these wake-state effects may alter subsequent sleep continuity and architecture.

Two human contexts are particularly relevant to this model. In pediatric epilepsy, ketogenic therapy may improve sleep quality not only through direct effects on sleep-regulatory pathways but also through improved seizure control, reduced nocturnal disruption, and better daytime attention or behavioral function [37]. In trained cyclists, ketone ester ingestion after strenuous evening exercise counteracted exercise-induced sleep disruption, including reduced REM sleep and increased wakefulness after sleep onset, suggesting that exogenous ketosis may support sleep when the preceding waking period imposes high metabolic and physiological stress [61]. More recently, Buga et al. reported that ketogenic precursor supplementation during four nights of experimental sleep restriction increased wearable-derived deep sleep by approximately 13% relative to placebo while also improving several waking performance measures [62]. Although sleep staging was derived from a wearable rather than PSG, these findings further support the possibility that ketone availability may modify sleep and waking performance under conditions of physiological sleep stress.

An unresolved question is whether the magnitude and timing of ketone exposure differentially influence waking function and subsequent sleep. Current human studies do not establish a dose-dependent spectrum in which ketones enhance daytime wakefulness at lower or daytime exposures but produce nocturnal hyperarousal at higher or nighttime exposures. Indeed, favorable waking and sleep effects can occur within the same intervention, as illustrated by improved waking performance together with increased wearable-derived deep sleep during experimental sleep restriction [62], while pre-sleep ketone administration has produced neutral or favorable sleep outcomes in other controlled studies [60,61]. These observations suggest that dose should be considered together with circadian timing, formulation, duration of exposure, feeding state, preceding sleep–wake history, and physiological stress rather than as an isolated determinant of sleep or wakefulness.

This framework shifts the question from whether ketones are “sleep-promoting” or “wake-enhancing” to how ketone exposure interacts with metabolic and neurophysiological states across the 24-h cycle. Future studies should therefore pair nocturnal EEG and blood ketone levels with daytime measures of alertness, fatigue, cognition, seizure burden, exercise recovery, autonomic tone, inflammatory markers, and ketone dynamics. Such designs would help distinguish direct effects of ketones on sleep-generating mechanisms from indirect effects mediated by improved wake-state function. The human studies discussed across these clinical and experimental contexts are summarized in Table 2.

## 6. Integrative Interpretation

Across preclinical and human studies, the direction and magnitude of ketone-associated sleep effects differ by intervention type, ketone species, formulation, species, sex, age, disease state, circadian timing, and sleep endpoint. This heterogeneity is not merely a limitation of the literature; it is central to the biology. Ketones appear to modify the metabolic and neurophysiological conditions under which vigilance states are generated, rather than acting as direct sleep-promoting agents in a uniform manner.

The most parsimonious interpretation of the current literature is therefore that ketones exert state- and context-dependent effects on sleep. In some models, ketogenic diets have been associated with increased NREM sleep or reduced REM sleep [47,50], whereas other studies report enhanced REM sleep in pediatric epilepsy and aged female rats [27,37]. In mice, a medium-chain triacylglycerol-containing ketogenic diet reduced REM duration [26], while in humans, ketone ester ingestion preserved REM sleep and improved sleep efficiency after strenuous evening exercise [61]. In contrast, nutritional ketosis did not improve subjective sleep, mood, or morning vigilance in healthy adults with presumably limited baseline sleep impairment [51]. These findings are not necessarily contradictory if ketones are understood as metabolic signals whose effects depend on sleep pressure, circadian timing, energetic stress, respiratory physiology, inflammation, disease state, and baseline sleep–wake stability.

A major mechanistic distinction is between AcAc and BHB. In the most direct preclinical sleep-homeostasis study, AcAc, but not BHB, increased slow-wave activity during NREM sleep after central administration in mice [6]. This finding aligns with mechanistic evidence that AcAc can suppress vesicular glutamate transport and reduce glutamate release [12], providing a plausible pathway through which AcAc could influence NREM sleep intensity. In contrast, BHB may influence sleep more indirectly through redox, inflammatory, epigenetic, and circadian pathways, including HDAC inhibition, histone β-hydroxybutyrylation, and suppression of NLRP3 inflammasome activation [7,8,9]. Thus, BHB and AcAc should not be treated as interchangeable in future sleep studies. Peripheral BHB is convenient to measure, but it may not capture the full ketone signal relevant to brain-state regulation. This limitation is especially relevant when comparing clinical formulations, because direct BHB preparations, ketone salts, ketone esters, fasting, and ketogenic diets can produce different temporal and relative exposures to BHB and AcAc despite all being described broadly as “ketosis.”

A second integrative theme is that ketones may influence sleep indirectly by improving the quality, stability, or recovery state of wakefulness. Sleep architecture is shaped not only by nocturnal processes but also by the metabolic and neurophysiological state established during the preceding waking period. This framework is particularly relevant in epilepsy, where ketogenic metabolic therapy may improve seizure control, reduce nocturnal epileptiform activity, improve daytime alertness, and stabilize neural networks, all of which may secondarily improve sleep continuity or architecture. Similarly, the Robberechts et al. exercise study suggests that ketone ester ingestion may improve sleep after a metabolically and physiologically demanding waking period, rather than acting as a conventional hypnotic [61]. These examples support a 24-h model in which ketones may improve subsequent sleep by altering wake-state stress, recovery physiology, energetic stability, or neural excitability.

Reviews have reached similarly cautious conclusions. Pasca et al. found heterogeneous but generally suggestive evidence for improved sleep quality and altered sleep structure under ketogenic dietary therapies [36]. O’Hearn proposed that ketogenic diets may support slow-wave sleep and circadian synchrony through metabolic stabilization mechanisms [76]. Masi et al. emphasized interactions among ketone bodies, appetite hormones, sleep timing, and circadian endocrine rhythms [28], while St-Onge et al. reviewed broader macronutrient effects on sleep architecture and sleep quality [77]. Together, these reviews support the conclusion that ketone biology is relevant to sleep, but that the clinical effects of ketogenic interventions depend on the population, intervention, and sleep phenotype being studied.

The clinical message is therefore not that ketones are hypnotics. Rather, ketogenic metabolic therapies and exogenous ketones may modulate sleep when baseline physiology creates a detectable target, such as pathological excitability, metabolic dysfunction, energetic stress, inflammation, sleep-disordered breathing, aging-related vulnerability, or recovery from strenuous waking activity. This conditional framework better explains the mixed literature than a simple model in which ketones universally deepen sleep, increase REM sleep, or improve subjective sleep quality.

## 7. Clinical Interpretation and Safety Considerations

For healthcare providers, ketogenic diets and exogenous ketone consumption should not be treated as interchangeable interventions. Ketogenic diets alter macronutrient intake, fiber consumption, micronutrient exposure, electrolytes, body water, insulin, leptin, thyroid and reproductive hormones, medication requirements, gastrointestinal patterns, body mass, and sometimes circadian feeding rhythms. Each of these variables can affect sleep independently of ketone bodies. Exogenous ketones, by contrast, acutely raise circulating ketones without necessarily reproducing the broader metabolic adaptation of fasting or ketogenic dietary therapy. Ketone esters, ketone salts, and direct BHB beverages also differ in pharmacokinetics, mineral load, gastrointestinal tolerability, acid–base effects, and AcAc/BHB exposure.

Clinicians may also associate ketone bodies with diabetic ketoacidosis, which should be clearly distinguished from nutritional or therapeutic ketosis. Nutritional ketosis and ketosis generated from exogenous sources generally occur in the setting of preserved physiological buffering and without the severe insulin deficiency, dehydration, electrolyte derangements, and metabolic acidosis that define ketoacidosis. This distinction is clinically important because neurological manifestations of ketoacidosis, including lethargy, somnolence, and impaired consciousness, should not be interpreted as evidence that therapeutic ketosis is inherently sedating or sleep-promoting.

A mechanistic anesthesiology study by Yang et al. reported that β-hydroxybutyric acid and acetone produced anesthetic-like effects in Xenopus tadpoles and modulated inhibitory receptor function in Xenopus oocytes at concentrations relevant to ketoacidosis or at substantially higher concentrations required for immobility [78]. This study is useful as a reminder that very high pathological ketone concentrations can interact with anesthetic-sensitive ion channels, including glycine and GABAA receptors. However, its relevance to ketogenic metabolic therapy and sleep is limited. The experiments were performed in Xenopus oocytes and tadpoles rather than mammalian in vivo sleep models; the anesthetic endpoints were immobility and ion-channel modulation rather than sleep architecture; and the concentrations required for anesthetic immobility were far above those typically achieved during nutritional ketosis or clinically studied exogenous ketone supplementation. Therefore, these data should be interpreted as supporting caution in ketoacidosis and other extreme ketotic states, not as evidence that nutritional ketosis acts as an anesthetic or direct hypnotic in humans.

Clinical interpretation should therefore be individualized by population and mechanism. In patients with metabolic disease or obesity, improvements in sleep quality during nutritional ketosis may reflect weight loss, improved glycemic control, reduced nocturia, reduced reflux, lower inflammatory tone, improved energy stability, or changes in medication exposure rather than ketosis itself. In neurological populations, sleep improvement may occur indirectly through reduced seizures, reduced migraine burden, improved fatigue, improved mood, or improved daytime function. In athletes or physically active individuals, exogenous ketones may influence sleep primarily under conditions of high recovery demand, such as strenuous evening exercise. These distinctions are important because they determine whether ketones should be conceptualized as sleep-stage modulators, recovery substrates, metabolic stabilizers, respiratory–metabolic interventions, or indirect contributors to better sleep–wake organization.

From a practical standpoint, clinicians should ask whether new insomnia, early-morning awakening, or subjective hyperarousal emerged after initiation or intensification of carbohydrate restriction, prolonged fasting, or pre-sleep ketone supplementation. At present, no sleep-optimal nocturnal BHB range or upper BHB threshold associated with hyperarousal has been established. Evening carbohydrate intake can alter sleep physiology in non-ketogenic individuals: a high-glycemic-index meal consumed four hours before bedtime shortened sleep-onset latency [79], whereas a high-carbohydrate bedtime supplement increased REM sleep during the first half of the night and decreased stage 4 sleep across the night, without affecting sleep onset [80]. However, these studies did not test ketogenic participants, measure nocturnal ketone suppression, or evaluate a bedtime carbohydrate intervention for ketogenic-diet-associated insomnia. Modification of evening carbohydrate intake or nocturnal ketosis should therefore currently be considered individualized clinical troubleshooting rather than an evidence-based sleep intervention. Prospective dose- and timing-focused studies incorporating nocturnal ketone monitoring and objective sleep assessment are underway and should help determine whether sleep responses track ketone exposure itself or the broader dietary and physiological context [81,82].

Safety considerations are especially important in clinical populations. Ketogenic diets and exogenous ketones require particular caution in individuals who use insulin, sulfonylureas, or sodium–glucose cotransporter-2 inhibitors; are pregnant or undergoing prolonged fasting; or have type 1 diabetes, insulin deficiency, acute illness, dehydration, alcohol use disorder, eating disorders, advanced kidney or liver disease, pancreatitis, disorders of fatty acid oxidation, or seizure disorders requiring medically supervised ketogenic therapy. Medication adjustment may be necessary when carbohydrate intake is substantially reduced, particularly in diabetes and epilepsy. Clinicians should also monitor tolerability, hydration, electrolyte balance, gastrointestinal symptoms, lipid responses, menstrual or endocrine effects, unintended weight loss, and symptoms suggestive of ketoacidosis. In high-risk contexts, nausea, vomiting, abdominal pain, tachypnea, confusion, unusual somnolence, or worsening fatigue should prompt clinical evaluation rather than being attributed to benign “ketone effects” or improved sleep.

Sleep-disordered breathing requires separate consideration. In obesity hypoventilation syndrome or obstructive sleep apnea, ketosis-related improvements in respiratory exchange, carbon dioxide production, nocturnal oxygenation, or apnea severity may be clinically useful. However, these effects should be integrated with standard sleep-medicine care rather than replacing evidence-based evaluation and treatment. Patients with suspected or untreated sleep apnea should still receive appropriate diagnostic testing and indicated therapies, including positive airway pressure when appropriate. Improvements in weight or nocturnal oxygenation during ketogenic therapy do not necessarily indicate direct normalization of sleep architecture or elimination of respiratory risk.

For both ketogenic diets and exogenous ketones, clinicians should avoid presenting ketones as universal sleep aids. A more appropriate message is that ketogenic metabolic therapy may improve sleep in selected contexts, particularly when sleep disruption is linked to metabolic dysfunction, neurological disease, inflammatory burden, respiratory physiology, or impaired recovery from wake-state stress. Clinical use should therefore include baseline sleep assessment, screening for sleep-disordered breathing when indicated, monitoring of daytime alertness and fatigue, and objective sleep testing when symptoms persist or when respiratory risk is present.

## 8. Gaps and Research Priorities

First, future human studies need to distinguish BHB from AcAc. Most clinical work measures peripheral BHB because it is convenient and commercially accessible, yet the strongest direct sleep-homeostasis signal points to AcAc as the ketone species that increases slow-wave activity during NREM sleep in mice [6]. Because BHB and AcAc are interconvertible and their relative abundance depends partly on redox state, BHB concentration alone may not adequately characterize biologically relevant ketone exposure. Trials should measure BHB and AcAc when feasible, along with glucose, lactate, free fatty acids, insulin, respiratory exchange, and nocturnal ketone dynamics. The AcAc/BHB ratio may provide complementary information about ketone interconversion and metabolic redox state, although its relationship to brain ketone exposure and sleep physiology remains to be established.

Second, studies should characterize nocturnal metabolic dynamics rather than relying only on daytime or fasting ketone measurements. A single morning BHB value may not reflect ketone exposure during sleep onset, early-night NREM sleep, REM-rich late-night sleep, or recovery from prior wake-state stress. Dose and timing should therefore be examined jointly. Future dose-ranging studies should compare daytime and pre-sleep ketone exposure while measuring both waking function and objective nocturnal sleep physiology to determine whether the same ketone concentration has different effects across circadian phase or vigilance state. Such studies should characterize achieved BHB and AcAc concentrations, exposure duration, meal and fasting state, circadian phase, and preceding sleep pressure to determine ketone concentration–response relationships. No human studies have established a ketone concentration associated with optimal sleep outcomes. Continuous interstitial ketone monitoring may help address this gap by capturing overnight BHB dynamics that are missed by isolated blood measurements. Current continuous ketone monitoring devices measure interstitial BHB rather than AcAc, and issues including sensor lag, drift, and validation during sustained nocturnal ketosis need to be considered [83,84]. Future work should combine polysomnography with EEG spectral analysis, repeated or continuous nocturnal ketone measurements, subjective sleep instruments, next-day vigilance and cognition testing, inflammatory markers, autonomic measures, respiratory monitoring, and circadian phase markers. Key sleep endpoints should include NREM slow-wave activity, REM timing, REM density, sleep spindles, arousal index, wake after sleep onset, sleep efficiency, oxygenation, carbon dioxide measures, and heart-rate variability.

The field is now beginning to address this gap by prospectively characterizing ketone dose and timing, nocturnal ketone kinetics, sleep physiology, and waking performance in several ongoing clinical trials. The Johns Hopkins K-SLEEP study (NCT07224074) [81] is evaluating two doses of pre-sleep 1,3-butanediol while obtaining repeated nocturnal BHB measurements and objective headband-EEG sleep assessments. The ongoing STAK-Sleep + Feed trial (NCT06075914) [82] uses a three-arm design comparing a ketogenic diet, a mixed/Mediterranean diet with placebo, and a mixed/Mediterranean diet with ketone ester supplementation during a standardized sleep-restriction challenge. Importantly, the protocol includes continuous interstitial BHB and glucose monitoring. Together, these studies should begin to define how ketone dose, temporal exposure, dietary context, and preceding sleep stress interact to influence sleep outcomes. Recent results from the related STAK-Sleep trial (NCT05519644) further illustrate this approach. During experimental sleep restriction, supplementation with a ketogenic precursor increased wearable-derived deep sleep while also improving selected waking-performance measures [62], although confirmation of the sleep-stage findings using EEG is still needed. These studies represent important steps toward defining sleep-specific therapeutic ketone concentrations.

Third, ketosis must be separated from energy deficit, weight loss, and broader dietary change. This will require isocaloric control diets, non-ketogenic caloric restriction controls, exogenous-ketone arms, ketone-matched designs, or factorial trials comparing ketogenic diet, non-ketogenic weight loss, and exogenous ketone exposure. In obesity, diabetes, and sleep-disordered breathing, sleep outcomes should be interpreted alongside changes in body mass, fat distribution, fluid balance, glycemic variability, medication exposure, airway mechanics, and ventilatory control. Patient-reported outcomes such as the Pittsburgh Sleep Quality Index are clinically valuable, but they cannot determine whether ketosis changes sleep architecture, EEG physiology, or respiratory events.

Fourth, studies should define the sleep challenge being tested. Ketones may have modest or undetectable effects in healthy good sleepers under baseline conditions, but may become more relevant during metabolic stress, inflammation, neurological disease, sleep deprivation, circadian disruption, strenuous exercise, aging, or impaired glucose metabolism. Future trials should specify whether ketones are being tested as direct sleep-stage modulators, circadian–metabolic signals, recovery substrates, respiratory–metabolic interventions, or therapies for disease-related sleep disruption. This distinction will improve trial design and reduce overgeneralization.

Fifth, epilepsy should remain a central translational model for ketone–sleep research. It is the most established human context for ketogenic metabolic therapy and provides a clinically meaningful setting in which ketone metabolism, neuronal excitability, sleep EEG, nocturnal epileptiform activity, seizure control, and wake quality intersect. Future epilepsy studies should incorporate baseline and follow-up sleep assessments, sleep EEG or polysomnography when feasible, interictal discharge quantification across vigilance states, standardized sleep questionnaires, daytime alertness and cognition measures, antiseizure medication tracking, and nocturnal ketone measurements.

Sixth, aging, sex, and developmental physiology differences deserve targeted study. Ketone production and cerebral ketone utilization differ across the lifespan, with children having a greater physiological capacity for ketogenesis and cerebral ketone utilization than adults, reflecting developmental differences in substrate demand, monocarboxylate transport, and ketolytic enzyme activity [85,86]. Developmental differences also extend to neural excitation/inhibition and stress-regulatory systems. GABAergic circuitry and receptor composition continue to mature through adolescence, and human spectroscopy studies demonstrate age-related changes in frontal GABA/glutamate balance [87,88]. Purinergic signaling, including adenosine A1-receptor expression, is also developmentally regulated, although much of this evidence is preclinical [89]. HPA-axis regulation changes across childhood and puberty, and metabolic responses to fasting are age-dependent; children can develop substantially higher BHB concentrations and different cortisol and counter-regulatory responses than adults during prolonged fasting [90,91]. However, none of these developmental differences has been shown to mediate age-dependent sleep responses to ketogenic therapy. Pediatric epilepsy studies have reported preserved slow-wave sleep and increased REM sleep during ketogenic therapy [37,38], but no study has directly compared pediatric and adult sleep responses at matched ketone exposure. Moreover, adult studies across healthy, neurological, metabolic, and psychiatric populations have not demonstrated a consistent pattern of ketone-associated insomnia. Thus, developmental stage is a plausible but currently unproven modifier of ketone–sleep physiology. At the other end of the lifespan, aged-rat data suggest that female REM sleep and hippocampal lipid-redox biology may be particularly responsive to ketone-based interventions [27]. Future studies should therefore compare ketone–sleep responses across the lifespan, including children, young adults, perimenopausal and postmenopausal women, older men, and individuals at risk for neurodegenerative disease, ideally using matched ketone exposure and sex-stratified analyses with hormonal status characterized when relevant. These studies should measure nocturnal BHB and AcAc together with objective sleep architecture, cognitive outcomes, autonomic and HPA-axis responses, inflammatory and oxidative-stress markers, and, where feasible, indices of excitatory/inhibitory and purinergic signaling, while carefully characterizing dietary fat source, ketone formulation, dose, and timing.

**Figure 1:**
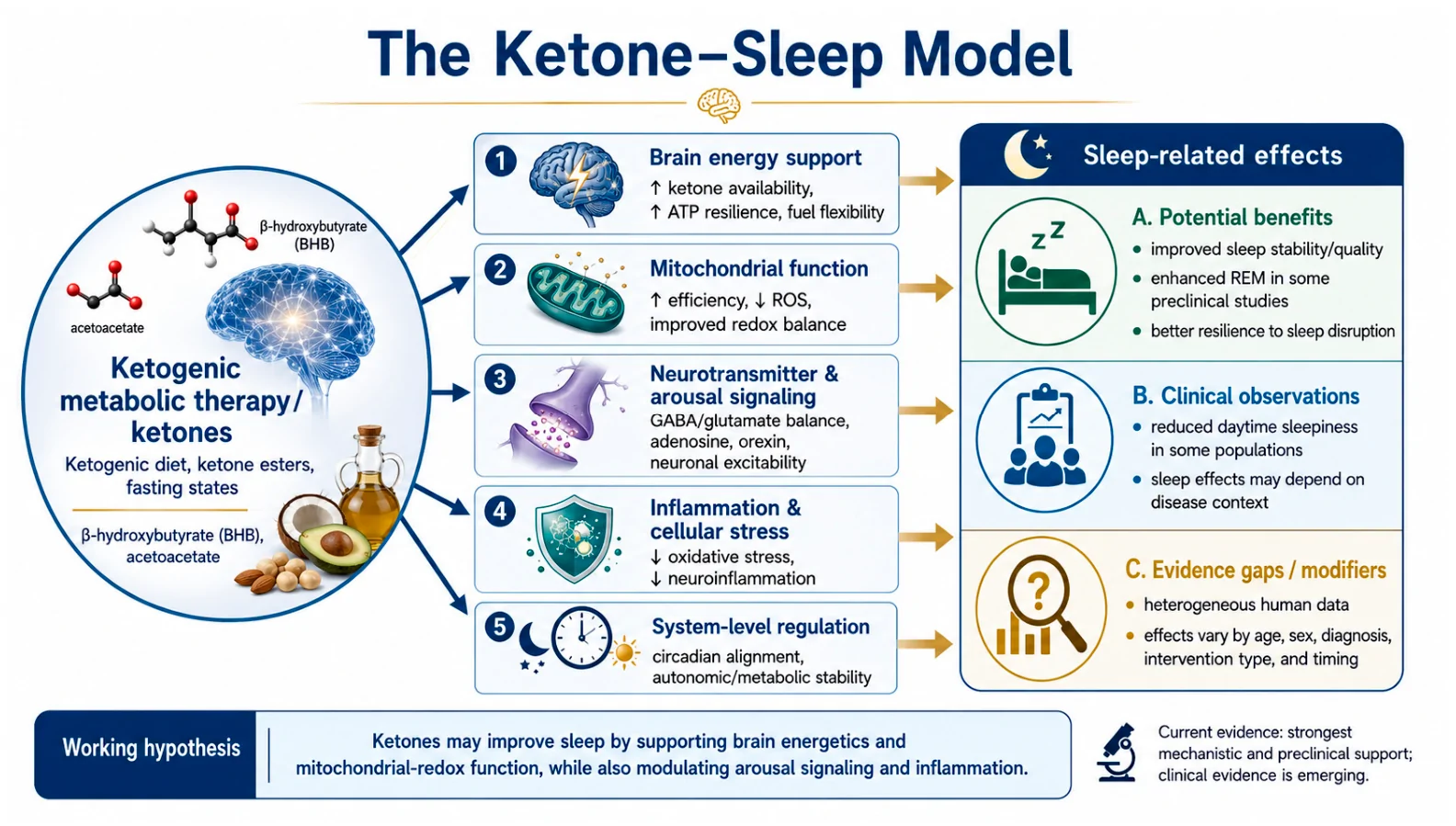
Working model for ketone–sleep interactions. Figure 1 summarizes the proposed model in which ketone bodies influence sleep through partially overlapping effects on substrate availability, neuronal excitability, inflammatory tone, redox biology, epigenetic regulation, circadian timing, and respiratory/metabolic physiology. The figure was created with assistance from ChatGPT-5.6 (OpenAI). All scientific content and labels were reviewed and verified by the authors.

Finally, future studies should assess wake-state quality as a mediator of sleep outcomes. If ketones improve daytime alertness, fatigue, seizure control, cognitive efficiency, exercise recovery, mood stability, or inflammatory tone, these wake-state effects may improve subsequent sleep continuity and architecture. Studies should therefore measure the entire 24-h sleep–wake cycle rather than treating nocturnal sleep as an isolated endpoint. These mechanistic pathways, clinical observations, and key modifiers are integrated in the working model shown in Figure 1.

## 9. Conclusions

Ketone bodies plausibly influence sleep through multiple mechanisms, but the strength and directness of evidence differs across pathways. The most direct preclinical evidence indicates that AcAc can increase slow-wave activity during NREM sleep and suppress glutamatergic signaling, suggesting a potential role in sleep homeostasis. BHB, in contrast, may influence sleep more indirectly through inflammatory, epigenetic, redox, circadian, and metabolic-stress pathways. These ketone species should therefore be separated mechanistically and experimentally.

Human evidence is promising but limited. Acute very-low-carbohydrate diets suggest that shifts in substrate availability can increase slow-wave sleep and alter REM sleep in some healthy adults. Ketogenic therapy can modify REM sleep, sleep EEG, daytime alertness, and subjective sleep quality in neurological populations, especially epilepsy, where effects may be mediated by reduced seizure burden and improved wake-state stability. In metabolic disease and obesity, improved sleep quality may occur during nutritional ketosis, but effects are difficult to disentangle from weight loss, glycemic improvement, reduced inflammation, and changes in sleep-disordered breathing. Exogenous ketone esters can improve sleep efficiency after strenuous evening exercise but may not overcome hypoxia-driven sleep disruption.

The field should therefore move away from generic claims that ketones improve sleep and toward mechanistically precise questions: which ketone species, at what concentration, at what circadian time, in which population, under which sleep challenge, and measured by which endpoint? Current evidence supports ketones as sleep-relevant metabolic signals whose effects are conditional, context-dependent, and likely mediated across the full 24-h sleep–wake cycle rather than solely through direct nocturnal sleep induction.

## Figures and Tables

**Table 1:** Mechanistic and preclinical evidence relevant to ketone–sleep biology.

Study	Model/Context	Ketone or Metabolic Exposure	Sleep-Relevant Finding	Interpretation/Limitation
Aalling et al. [1]	Sleep-state brain energetics review	Sleep-dependent substrate shifts	Ketone and fatty-acid-derived substrate oxidation may partly replace glucose during sleep.	Supports plausibility of state-dependent ketone actions.
Chikahisa et al. [6]	Mouse sleep-deprivation model; central AcAc vs. BHB	6 h sleep deprivation; intracerebroventricular ketone administration	Sleep deprivation increased circulating ketones and AcAc/BHB ratio; AcAc, not BHB, increased NREM SWA and suppressed glutamate release.	Strongest direct sleep-homeostasis evidence; central injection limits translation.
Kondo et al. [23]	PPAR-alpha knockout vs. wild-type mice	24 h food deprivation	PPAR-alpha deficiency produced lower ketone responses to fasting and reduced ability to maintain wakefulness late in the dark phase.	Links fasting ketogenesis/lipid signaling with vigilance-state regulation; not a ketone-only manipulation.
Danguir et al. [22]	Lean and obese rat starvation models	Food deprivation/starvation	Starvation altered slow-wave sleep differently by adiposity/metabolic state.	Fasting studies conflate ketosis with stress, caloric deficit, and endocrine changes.
Westmark et al. 2023 [24]	Wild-type and Fmr1 knockout mice	Ketogenic diet from early life	Ketogenic diet changed sleep architecture with effects on NREM/REM distribution and circadian sleep measures.	Developmental model; genotype and diet-composition effects complicate translation.
Westmark et al. 2024 [25]	Adult C57BL/6J wild-type and Fmr1 knockout mice	Adult-onset ketogenic diet	Adult ketogenic diet increased NREM sleep during the dark cycle; some genotype-specific sleep features persisted.	Supports adult-onset diet effects; mechanisms remain incompletely resolved.
Zhuang et al. [26]	Mouse medium-chain triglyceride (MCT) ketogenic diet model	MCT-containing ketogenic diet	MCT-containing ketogenic diet reduced REM sleep duration without major circadian disruption.	Demonstrates that dietary fat source and formulation may change the REM signal.
Lark et al. [27]	Aged Fischer-344 rats, both sexes	Ketone ester gavage or 2 months on the ketogenic diet	Ketone ester and ketogenic diet increased REM sleep in aged females; ketogenic diet improved novel place recognition in both sexes and reduced hippocampal lipid peroxidation in females.	Important aging/sex-difference study; translational implications need human testing.

Abbreviations: AcAc, acetoacetate; BHB, β-hydroxybutyrate; Fmr1, fragile X messenger ribonucleoprotein 1; MCT, medium-chain triglyceride; NREM, non-rapid eye movement; PPAR-alpha, peroxisome proliferator-activated receptor alpha; REM, rapid eye movement; SWA, slow-wave activity.

**Table 2:** Human studies linking ketogenic interventions or ketone exposure with sleep outcomes.

Study	Population	Ketotic Context	Sleep Endpoint	Main Finding	Key Limitation
MacFadyen et al. [47]	Young men	~4 days of starvation	EEG sleep staging	Increased stage 3–4/SWS and reduced REM reported with reversal after refeeding.	Fasting confounds ketosis with caloric deficit and stress.
Phillips et al. [48]	8 healthy young men	Isocaloric macronutrient manipulation	EEG sleep staging	Diet-dependent SWS and REM changes; low-carbohydrate/high-fat condition differed from balanced diet.	Ketones not measured; small sample.
Kwan et al. [49]	Healthy adult women	1 week 50 g/day carbohydrate diet	Ambulatory EEG + questionnaire	Plasma BHB increased; REM latency decreased, while total sleep and stage distribution were largely unchanged.	Short duration and small sample; ambulatory EEG.
Willi et al. [67]	Adolescents with morbid obesity	8 weeks ketogenic diet	EEG sleep staging	Weight loss was accompanied by improved sleep-abnormality measures and REM/non-REM indices.	Small sample; sleep effects likely mediated partly by weight loss.
Peng et al. [42]	7 patients diagnosed with febrile infection-related epilepsy syndrome	Enteral KD and intravenous KD	EEG sleep staging	Seizure burden and seizure duration reduced. Sleep architecture normalized.	Small sample size and two different KD methods (enteral and intravenous)
Reyes et al. [41]	12 patients with epileptic encephalopathy with electrical status epilepticus during slow sleep (ESES)	Hopkins protocol KD	EEG sleep staging before, during, and after KD treatment.	Pre-KD asymmetric bilateral spike-and-wave paroxysms in the EEG readings were reduced.	Age of KD treatment could correspond to spontaneous improvement of ESES.
Wu et al. [40]	52 children with pharmacoresistant epileptic encephalopathy	Hopkins protocol KD	Fully digital video EEG (VEEG) monitoring	Improved background rhythms and reduced spike wave index	Not a randomized trial
Robberechts et al. [61]	10 well-trained cyclists	Post-exercise and pre-bed ketone ester	Overnight PSG + EEG	KE improved sleep efficiency and counteracted exercise-associated REM loss.	Small athletic sample; context-specific.
Afaghi et al. [50]	14 healthy non-obese men	48 h very-low-carbohydrate diet	PSG	SWS percentage increased and REM percentage decreased compared with mixed diet.	Urine ketones; male good sleepers; acute adaptation only.
Hallbook et al. [37]	Children with therapy-resistant epilepsy	Therapeutic KD for 3–12 months	Ambulatory PSG	REM increased, SWS was preserved, and increased REM correlated with quality-of-life measures.	Seizures and medication changes are major confounders.
Osman et al. [66]	20 adults with obesity hypoventilation syndrome	2 weeks KD in a crossover sequence	PSG, gas exchange	Ketones rose to ~2 mM; CO_2_, apnea severity, and nocturnal oxygenation improved with rebound after regular diet.	Non-randomized and short; weight/fluid shifts may contribute.
Hallbook et al. [38]	18 children with therapy resistant epilepsy	Classical KD after 12 h fast	24 h ambulatory EEG monitoring. 1 week prior and after 3 months of KD	Interictal epileptiform discharges reduced during NREM Stage 2, SWS and REM	The children received slightly different diets and were supplemented with vitamins and other substances.
Dressler et al. [75]	Infants with drug-resistant infantile epileptic spasms syndrome	KDT or ACTH as sequential treatment	Epilepsy/EEG outcomes	Retained as pediatric epilepsy/EEG context; KDT was part of treatment sequencing.	Not a general sleep-architecture trial; sleep relevance is indirect.
Iacovides et al. [51]	11 healthy normal-weight adults	3-week nutritional ketosis vs. HCLF diet	PSQI, mood, cognition	No sleep, mood, or cognition differences despite confirmed nutritional ketosis.	Small healthy cohort; subjective sleep only.
Sethi et al. [58]	23 adults with bipolar disorder or schizophrenia	4-month ketogenic diet therapy	PSQI	19% showed enhanced sleep quality	Psychiatric population, subjective sleep measurement
Siegmann et al. [63]	Type 2 diabetes/prediabetes	1-year continuous care nutritional ketosis	PSQI	Patient-reported sleep improved and fewer participants were poor sleepers.	Multicomponent intervention: weight, glucose, and behavior changed.
Barrea et al. [65]	324 women with overweight/obesity	31-day active VLCKD phase	PSQI, body composition, hs-CRP	PSQI improved; sleep-quality change was largely predicted by fat-mass reduction.	Open-label uncontrolled design; weight/fat-loss mediation likely.
Pasca et al. [56]	Pediatric migraine patients	3-month KD	PSQI and objective sleep measures	Preliminary prospective data suggested sleep effects, with mixed results across endpoints.	Small preliminary cohort; disease improvement and adherence may confound effects.
Merlino et al. [53]	21 patients with relapsing–remitting MS	6-month KD	PSQI and ESS	PSQI and ESS scores improved after KD; sleep quality contributed to quality of life/psychological benefits.	Exploratory cohort; subjective sleep.
Tereshko et al. [57]	Adults with chronic migraine	Prospective KD	PSQI and ESS	Subjective sleep quality and daytime sleepiness improved with migraine outcomes.	No objective sleep physiology; symptom improvement may mediate sleep benefit.
Merlino et al. [55]	Migraine cohort	Clinical KD	PSQI and ESS	Sleep improved during KD, apparently independent of pain-control effects.	Subjective sleep endpoints; disease-activity confounding possible.
Perlman et al. [54]	65 people with relapsing MS	6-month KD with follow-up at 1, 3, and 6 months	ESS and sleep-disorder symptom checklist	Fewer positive screens for insomnia, OSA, and restless legs syndrome were reported after KD.	Subjective screening, no PSG.
Katsuya et al. [60]	90 healthy Japanese adults (30/group)	1.5 g or 2.9 g D-BHB for 14 days	OSA-MA sleep inventory	D-BHB improved subjective sleep quality domains including sleepiness on rising, dreaming, maintenance, and refreshment.	Subjective endpoints; achieved nocturnal ketones not fully characterized.
Stalmans et al. [71]	11 healthy men at simulated altitude	Post-exercise/pre-sleep ketone ester	PSG, oxygenation, blood gases	Hypoxia reduced sleep efficiency/SWS; KE improved severe desaturation but not architecture.	Small sample; altitude physiology may overwhelm ketone effects.
Husain et al. [52]	9 patients with narcolepsy	Low-carbohydrate ketogenic diet for 8 weeks	Narcolepsy symptom score	Symptoms improved by approximately 18%.	Uncontrolled, small, questionnaire-based.
Buga et al. [62]	32 healthy adults (16 women)	25 g bis-octanoyl (R)-1,3-butanediol ketogenic precursor vs. isocaloric placebo during 4 nights of 50% sleep restriction	Wearable-derived sleep duration and architecture (REM, NREM, deep sleep)	Ketogenic precursor increased deep sleep by 13% vs. placebo during sleep restriction while also improving waking performance.	Sleep staging was wearable-derived rather than PSG/EEG; experimental sleep restriction limits generalization to habitual sleep.

Abbreviations: ACTH, adrenocorticotropic hormone; BHB, β-hydroxybutyrate; CO_2_, carbon dioxide; D-BHB, D-β-hydroxybutyrate; EEG, electroencephalography; ESES, electrical status epilepticus during sleep; ESS, Epworth Sleepiness Scale; HCLF, high-carbohydrate, low-fat diet; hs-CRP, high-sensitivity C-reactive protein; KD, ketogenic diet; KDT, ketogenic dietary therapy; KE, ketone ester; MS, multiple sclerosis; OSA, obstructive sleep apnea; OSA-MA, Oguri–Shirakawa–Azumi Sleep Inventory, Middle-Aged and Aged version; PSG, polysomnography; PSQI, Pittsburgh Sleep Quality Index; REM, rapid eye movement; SWS, slow-wave sleep; VEEG, video electroencephalography; VLCKD, very-low-calorie ketogenic diet.

## Data Availability

No new data were created or analyzed in this study. Data sharing is not applicable to this article.

## Supplementary Materials

The following supporting information can be downloaded at: https://www.mdpi.com/article/10.3390/nu18203437/s1, Figure S1: Simplified study-selection flow diagram summarizing the literature identification and selection process used for this narrative review. The primary PubMed search identified 329 records through September 2026. Additional relevant sources were identified through targeted mechanistic searches, reference-list screening, and ClinicalTrials.gov. Because this was a narrative review and the supplementary search process was iterative, detailed PRISMA-style screening counts were not prospectively maintained. The final review cited 91 references in total, including 89 published sources and 2 ClinicalTrials.gov records. The core evidence summarized in the evidence tables comprised 8 mechanistic/preclinical items and 26 human studies. The figure was created with assistance from ChatGPT (OpenAI). All scientific content and labels were reviewed and verified by the authors.
