# Supplementary material for: Ketones and Sleep: A Narrative Review of Mechanisms, Preclinical Models, and Human Evidence
Source: Nutrients. 2026 Oct 20;18(20):3437. doi: 10.3390/nu18203437 (PMC13708312; doi:10.3390/nu18203437)
Supplement: Supplementary file 1 [file nutrients-18-03437-s001.zip › nutrients-4506050-supplementary.pdf]

Supplementary Materials

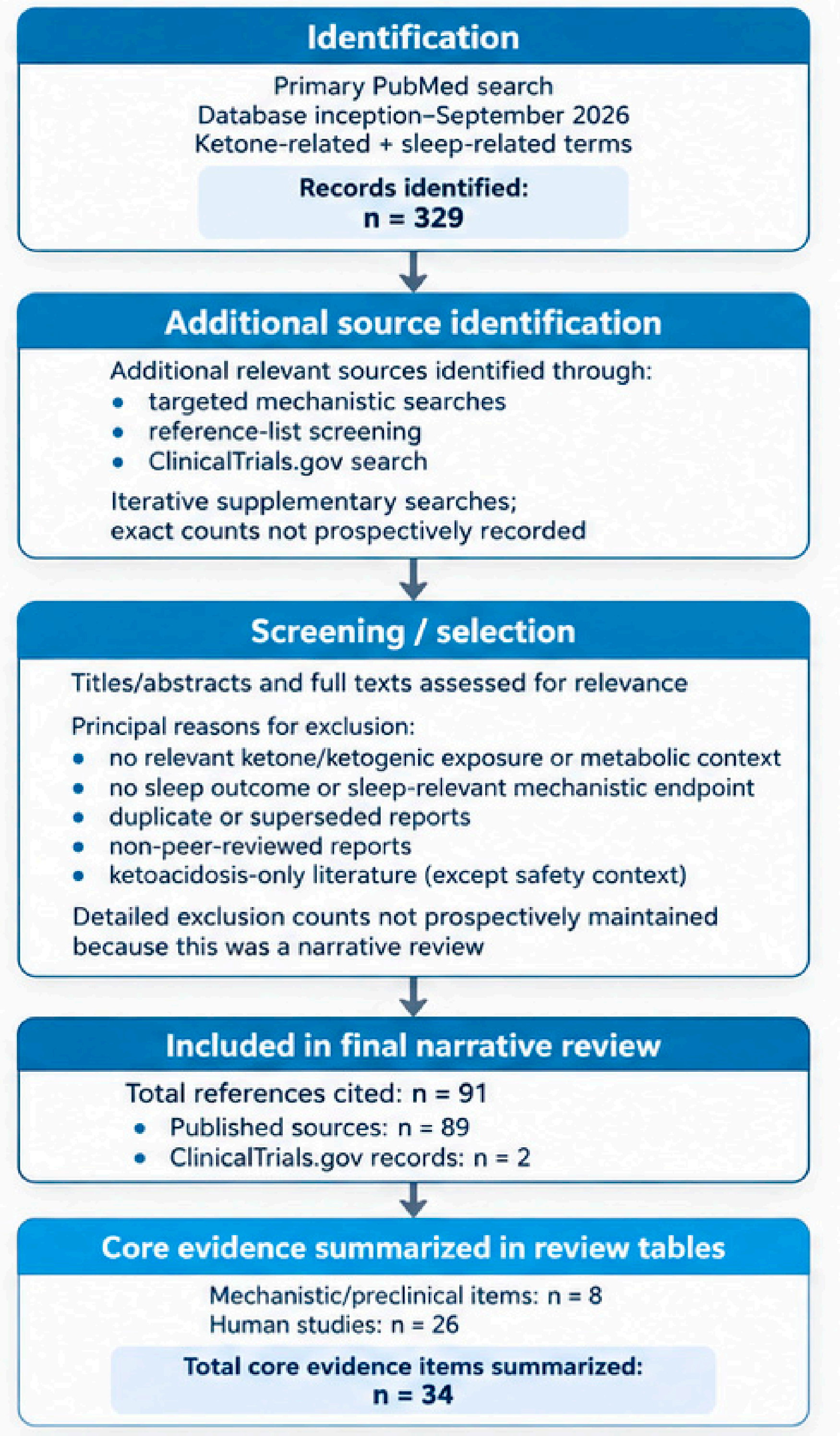

Figure S1. Simplified study-selection flow diagram summarizing the literature identification and selection process used for this narrative review. The primary PubMed search identified 329 records through September 2026. Additional relevant sources were identified through targeted mechanistic searches, reference-list screening, and ClinicalTrials.gov. Because this was a narrative review and the supplementary search process was iterative, detailed PRISMA-style screening counts were not prospectively maintained. The final review cited 91 references in total, including 89 published sources and 2 ClinicalTrials.gov records. The core evidence summarized in the evidence tables comprised 8 mechanistic/preclinical items and 26 human studies. The figure was created with assistance from ChatGPT (OpenAI). All scientific content and labels were reviewed and verified by the authors.
